# Differential Responses to Wnt and PCP Disruption Predict Expression and Developmental Function of Conserved and Novel Genes in a Cnidarian

**DOI:** 10.1371/journal.pgen.1004590

**Published:** 2014-09-18

**Authors:** Pascal Lapébie, Antonella Ruggiero, Carine Barreau, Sandra Chevalier, Patrick Chang, Philippe Dru, Evelyn Houliston, Tsuyoshi Momose

**Affiliations:** Sorbonne Universités, UPMC Univ Paris 06, and CNRS, Laboratoire de Biologie du Développement de Villefranche-sur-mer, Observatoire Océanographique, Villefranche-sur-mer, France; New York University, United States of America

## Abstract

We have used Digital Gene Expression analysis to identify, without bilaterian bias, regulators of cnidarian embryonic patterning. Transcriptome comparison between un-manipulated *Clytia* early gastrula embryos and ones in which the key polarity regulator Wnt3 was inhibited using morpholino antisense oligonucleotides (Wnt3-MO) identified a set of significantly over and under-expressed transcripts. These code for candidate Wnt signaling modulators, orthologs of other transcription factors, secreted and transmembrane proteins known as developmental regulators in bilaterian models or previously uncharacterized, and also many cnidarian-restricted proteins. Comparisons between embryos injected with morpholinos targeting Wnt3 and its receptor Fz1 defined four transcript classes showing remarkable correlation with spatiotemporal expression profiles. Class 1 and 3 transcripts tended to show sustained expression at “oral” and “aboral” poles respectively of the developing planula larva, class 2 transcripts in cells ingressing into the endodermal region during gastrulation, while class 4 gene expression was repressed at the early gastrula stage. The preferential effect of Fz1-MO on expression of class 2 and 4 transcripts can be attributed to Planar Cell Polarity (PCP) disruption, since it was closely matched by morpholino knockdown of the specific PCP protein Strabismus. We conclude that endoderm and post gastrula-specific gene expression is particularly sensitive to PCP disruption while Wnt-/β-catenin signaling dominates gene regulation along the oral-aboral axis. Phenotype analysis using morpholinos targeting a subset of transcripts indicated developmental roles consistent with expression profiles for both conserved and cnidarian-restricted genes. Overall our unbiased screen allowed systematic identification of regionally expressed genes and provided functional support for a shared eumetazoan developmental regulatory gene set with both predicted and previously unexplored members, but also demonstrated that fundamental developmental processes including axial patterning and endoderm formation in cnidarians can involve newly evolved (or highly diverged) genes.

## Author Summary

The recent wave of genome sequencing from many species has revealed that most of the gene families known to regulate animal development are shared not only between humans and laboratory favorites such as mice, flies and worms, but also by evolutionarily more distant animals such as jellyfish and sponges. It is often assumed that genes inherited from a common ancestor remain largely responsible for regulating embryogenesis across these animal species, rather than more recently evolved genes. To address this issue we made an unbiased, systematic search for developmental genes in embryos of the jellyfish *Clytia*, selecting genes whose expression altered upon manipulation of the key regulator Wnt3, and comparing their expression in embryos specifically disrupted for Planar Cell Polarity. Identification of evolutionarily conserved and novel genes as developmental regulators was confirmed by demonstrating characteristic expression profiles for a sub-set of genes, and by gene knockdown studies. Conserved genes coded for members of many known signaling pathway and transcription factor families, as well as previously unstudied proteins. Nearly 30% of the identified genes were restricted to cnidarians (the jellyfish-sea anemone-coral group), supporting the idea that the appearance of new genes during evolution contributed significantly to generating animal diversity.

## Introduction

A major challenge in biology is to understand how the current extraordinary diversity of animal forms has been generated during evolution. Specific goals are to determine which genes were employed to regulate developmental processes in the earliest multi-cellular animals, and how this set of regulators was expanded during the evolution of different animal branches by diversification of existing gene families or by the acquisition of new genes. To address these questions requires identification and functional analysis of developmental regulatory genes in species from right across the animal kingdom, covering not only the “bilaterian” (protostome plus deuterostome) branch including the classic experimental models such as mouse, zebrafish, *Drosophila* and *Caenorhabditis*, but also non-bilaterian phyla such as cnidarians, ctenophores and sponges, which have evolved many distinct forms and body plans.

Following the recent explosion of genome and transcriptome sequencing it has been widely noted that the majority of families of transcription factors and signaling pathway components uncovered as developmental regulators in bilaterian model species are represented in genomes of cnidarians, as well as ctenophores and to a lesser extent sponges [1]–[9]. This has fuelled the idea that a shared set (or “common toolkit”) of genes inherited from a common metazoan ancestor is used to regulate development in widely divergent species through differential deployment [1], [3], [10]–[17]. The common toolkit idea relies heavily on the assumption that conserved genes have retained largely equivalent developmental functions during the evolution of each animal lineage, for which evidence remains quite patchy. Comparing the expression territories, and in some cases functions, of gene orthologs in families of transcription factors such as Hox, Sox, Fox and T-box genes, and components in signaling pathways such as Wnt, TGFβ, FGF, Hedgehog, Notch etc, has provided some support for this assumption, but also for lineage specific modifications in gene repertoires for example through gene duplications and losses within the transcription factor families [10]–[21]. Another possibility is that novel regulatory genes have emerged within specific evolutionary lineages to contribute to generating animal diversity [14], [18]–[23]. The significant proportions of cnidarian-specific gene sequences in the fully sequenced genomes of *Hydra* (around 15%) and *Nematostella* (around 13%) is compatible with such a scenario in Cnidaria [14], [18], [22]–[26]. Detailed studies involving transcriptome comparisons in *Hydra* have shown that many cnidarian-specific genes are associated with specialized cell types, notably nematocytes (stinging cells) but also nerve and gland cells [22], [24]–[30], while others have been specifically implicated in intercellular signaling and regulating morphological processes [22], [27]–[31]. Furthermore, in a subtractive hybridization search for cnidarian-specific genes involved in embryogenesis, 30 of 88 distinct partial cDNA clones recovered did not match known bilaterian sequences, including one corresponding to a *Hydra* specific gene (HyEMB-1) expressed in the ovary and early embryo [31].

To gain a fresh perspective on the gene repertoires that regulate metazoan development, we employed a systematic unbiased comparative transcriptomics approach to identify potential regulators of embryonic patterning at gastrula stage in the cnidarian experimental model *Clytia hemisphaerica* [32]. Clytia is a typical hydrozoan species that includes a jellyfish form as well as a polyp form in its life cycle, unlike anthozoan cnidarians such as the popular sea anemone model *Nematostella vectensis*. After gastrulation, a torpedo-shaped “planula” larva is formed, whose organization shows the characteristic cnidarian body plan: a single “oral-aboral” axis and two germ layers. The outer ectoderm of the *Clytia* planula features ciliated epitheliomuscular cells for motility, and an internal endodermal (or “entodermal”) region including a population of interstitial stem cells (i-cells) specific to hydrozoans, which generate a variety of cell types for each germ layer [33]–[36]. Gastrulation proceeds by unipolar cell ingression to fill the blastocoel prior to endoderm cell epithelialization [37]. The gastrulation site derives from the egg animal pole and corresponds to the pointed oral pole of the larva, giving rise after metamorphosis to the mouth region of the polyp form [38].

Establishment of the oral pole in *Clytia* critically depends on Wnt/Fz signaling activity through the Wnt/β-catenin pathway. Maternally-provided transcripts for the ligand Wnt3 and the receptors Fz1 (activatory) and Fz3 (inhibitory) are pre-localized along the egg animal-vegetal axis to drive activation of this pathway on the future gastrulation site/oral side during cleavage and blastula stages [39], [40]. This activation establishes distinct regional identities characterized by specific sets of transcribed genes at the oral and aboral poles of the developing embryo, including those required for cell ingression at gastrulation. Fz-PCP signaling, dependent on the conserved transmembrane protein Strabismus (Stbm), is activated in parallel along the same axis to coordinate cell polarity in the ectoderm and to guide embryo elongation [41]. Since multi-member Wnt families with early polarized embryonic expression have also been uncovered in other cnidarians [42], [43], ctenophores and sponges [44]–[47] as well as in a range of bilaterian models [48], [49], it seems highly probable that Wnt/Fz signaling regulated embryonic patterning in ancestral metazoans, specifying the primary body axes and/or presumptive germ layer regions.

To identify genes potentially involved in *Clytia* embryogenesis without favoring gene families identified as developmental regulators from bilaterians, we compared transcriptomes at the onset of gastrulation between normal embryos and ones strongly “aboralized” by Wnt3 morpholino (Wnt3-MO) injection prior to fertilization [40]. In many animals gastrulation coincides with, or closely follows, a significant stepping up of transcription from the zygotic genome, taking over from an initial phase of development predominantly dependent on maternally supplied mRNAs and proteins. By comparing transcriptomes from undisturbed and Wnt3-MO early gastrulae by Digital Gene Expression (DGE) we compiled lists of significantly over- and under-expressed genes. These included orthologs of known conserved developmental regulators but also members of unexplored metazoan conserved gene families, and in addition many sequences restricted to cnidarians. Expression profiling for an unbiased subset of these transcripts systematically revealed spatially or temporally restricted expression profiles of four types. Further transcriptome and *in situ* hybridization comparisons with Fz1-MO and Stbm-MO embryos revealed expression-pattern-related differences in the responses of genes to disruption of Wnt/β-catenin versus PCP. Finally, roles in developmental processes for the identified genes, both conserved and cnidarian–restricted, were supported both by their characteristic expression patterns and by correlated phenotypes obtained following morpholino injection for a subset of 8 genes.

Overall our unbiased screen allowed systematic identification of developmental genes regulated by the Wnt/ß-catenin pathway and by Fz-PCP. It provided functional support for a shared eumetazoan developmental regulatory gene set with both predicted and previously unexplored members, while also showing that axial patterning and endoderm formation in cnidarians can involve taxon restricted genes.

## Results

### A systematic approach to identify cnidarian developmental genes

**Figure 1:**
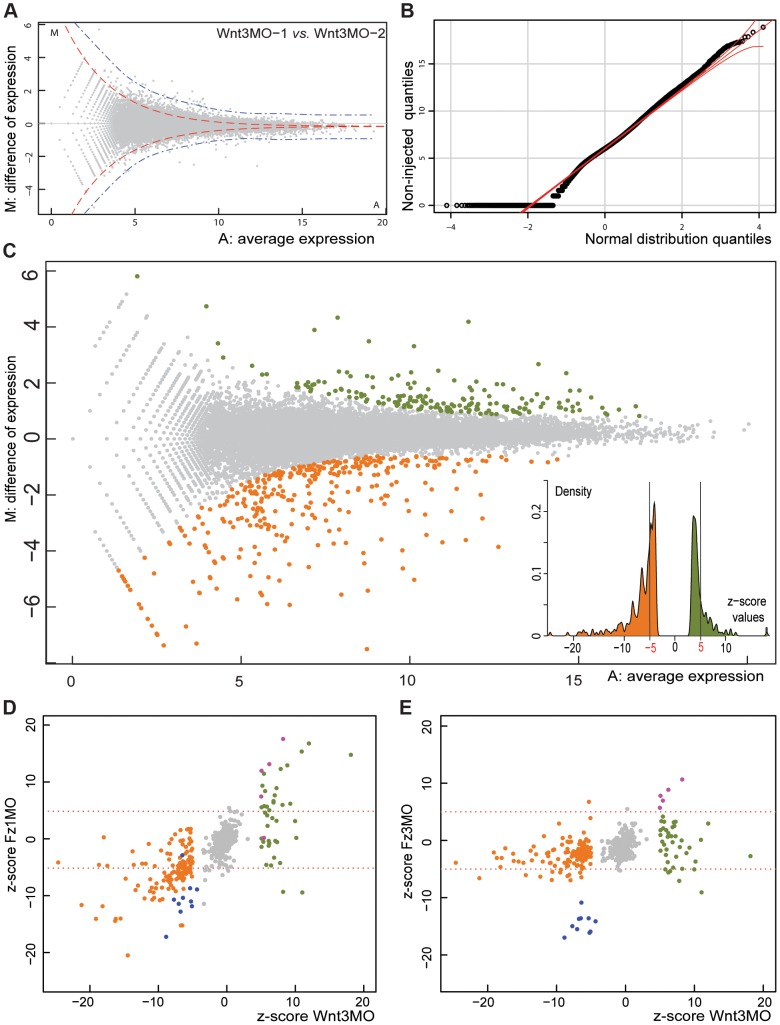
Identifying Clytia early gastrula patterning genes by DGE. A) Visualization of variation between two independent Wnt3-MO early gastrula replicate samples (“MA plot”). For each reference transcript (grey dots), M is the difference of read density between the two samples and A its average expression level in the combined samples (see Methods). Red and blue dotted lines give estimates of the distribution zone outside which values are statistically significant, based on the standard deviation (4xSD) of a theoretical distribution (red) calculated using the random sampling model, or real variation (blue) estimated by the comparison of technical replicates [50]. B) Demonstration of the near-normal distribution of the log_2_ counts of the mapped reads from one of the non-injected embryo samples by a QQ plot, a necessary condition to use the Random Sampling Model assumption in DGE analysis. C) MA plot of read data from Wnt3-MO versus non-injected embryo samples. Applying the 1% cut-off p-value for statistical significance corresponding to the threshold z-score of +/−3.3 identifies 148 transcript sequences as over-expressed (green dots) and 232 as under-expressed (orange dots) in Wnt3-MO embryos. The more stringent +/−5.0 threshold for z-score values eliminates a cluster of genes with expression characteristics very close to the overall population of non-differentially expressed genes, as demonstrated in the histogram (insert) and reduces the number of transcripts to 44 and 135 for Wnt3-MO embryo over- and under-expressed transcripts respectively. D, E) Z-scores for the Wnt3-MO embryo over- (green dots) and under- (orange dots) expressed transcripts plotted against those for two other morpholino-injected embryo groups harvested at the same developmental stage. Z-scores were calculated for experimental versus non-injected values in each case. D: Wnt3-MO versus Fz1-MO; E: Wnt3-MO versus Fz3 MO. Transcripts significantly under-expressed in all three MO groups (z-scores less than -5: probably from bacterial contaminants) are represented as blue dots and over-expressed (z-scores greater than 5; probably injection damage-related) as purple dots.

To identify genes regulated transcriptionally in relation to Wnt dependent embryo patterning we compared transcriptomes from unmanipulated early gastrula stage embryos and from embryos injected prior to fertilization with a morpholino antisense oligonucleotide targeting Wnt3 [40]. Digital Gene Expression analysis (DGE) was performed using an Illumina HiSeq sequencing platform. The number of mapped reads onto a reference transcriptome data set was taken as a measure of transcript level, and the statistical significance of differences in these levels between samples assessed using the DEGseq package (Figure 1; see Materials and Methods for technical details). Plotting for each transcript the expression ratio between two samples against the global average expression (Figure 1A,C) allowed visualization of sets of transcripts that showed significant differential expression, defined as ones that cannot be accounted for by sampling variation according to Random Sampling Model. We used the MATR method [50], justified by the Normal distribution of the data (Figure 1B), to adjust the cutoff to take into account experimental noise, based on comparison of replicate samples (blue line in Figure 1A: compare with the red line delimiting the theoretical random distribution). For subsequent analyses we routinely used a corresponding “z-score” value as an index of significant differences between samples (see Methods).

Comparisons between Wnt3-MO and uninjected embryo samples (Figure 1C) identified 375 assembled transcript sequences as differentially expressed according to the z-score +/−3.3 cutoff, which corresponds to a probability threshold (p-value) of 0.01 (colored dots in Figure 1C). Detailed analyses were performed for a more restricted set of 179 sequences with z-scores of less than -5 or greater than +5 (see insert in Figure 1C; list of transcripts and their characteristics in File S1). We could eliminate transcripts whose expression levels were affected non-specifically by the morpholino injection procedure by comparing the Wnt3-MO embryo differentially regulated transcripts with those identified in embryo populations generated using morpholinos targeting two other genes, Fz1 and Fz3, which respectively activate and repress Wnt/β-catenin signaling leading to aboralized and oralized phenotypes, respectively [39]. Genes non-specifically affected by the morpholino injection procedure are expected to respond in the same way in all three experimental groups, whereas genes regulated specifically downstream of Wnt3 are expected to respond distinctly following Fz1-MO compared to Fz3-MO injection. Comparison between these groups allowed us to identify 4 sequences with high z-scores (>5) in Fz1-MO and in Fz3-MO (opposite phenotypes) as well as Wnt3-MO samples (purple dots in Figure 1D,E; DGE class 5 in File S1). Two of these code for Ubiquitin ligases, implicated in protein degradation, and one for a secreted cyclase, suggesting a possible association with lysis of damaged cells in injected embryos. In addition the Fz3 transcript was itself detected at high levels in Fz3-MO embryos, probably due to the stabilizing effect of the morpholino. An additional set of 10 transcripts were eliminated as coming from likely bacterial contaminants, because they clearly stood apart as strongly under-represented (z-scores <−5) in both Fz3-MO and Wnt3-MO samples (and also for Fz1-MO in 9 cases) compared with uninjected controls (blue dots in Fig 1D, E.). The sequences of these transcripts had no similarity with any known eukaryotic genes but rather included genes from bacteria. Contamination from bacteria may be higher in uninjected embryos due to reduced manipulation of the egg and thus more frequent retention of the jelly coat and associated contaminants.

### Conserved and novel transcripts are differentially expressed in Wnt3-MO embryos

After elimination of the 13 non-specifically affected sequences, our final validated transcriptome comprised 166 differentially expressed transcripts. 153 of these 166 had clear predicted full or partial ORFs, comprising 40 over-expressed in Wnt3-MO embryos and 114 under-expressed. Detailed analysis of these sequences (File S1) revealed conserved and novel genes.

#### Conserved developmental regulators

*Clytia* developmental regulatory genes already known to be expressed in a polarized manner were present as expected. These included the orally expressed Brachyury (Bra), Frizzled-1 (Fz1) and WntX1A in the Wnt3-MO under-expressed list, and the aborally expressed FoxQ2a, Frizzled-3 (Fz3), Hox9/14B and Sox15 in the over-expressed list [15]–[17], [39], [40]. Many additional *Clytia* orthologs of bilaterian developmental regulators were also identified (phylogenetic analyses in File S2). Amongst the transcription factors were an ortholog of the hydrozoan-duplicated T-box Brachyury gene Bra2 [11], two forkhead family proteins frequently associated with endoderm formation (FoxA, FoxC), a previously uncharacterized FoxQ2 paralog (FoxQ2c), a T-box transcription factor (Tbx: no clear orthology to vertebrate T box genes), a member of the Pox-neuro branch of the Pax family (PaxA) [10], orthologs of Six4/5 and *Nematostella* DMRT-E [51], the Ets transcription factor Erg and the ANTP family non-hox/parahox homeodomain protein HD02 [16]. We also identified a Myb transcription factor belonging to the HTH class and several zinc finger domain transcription factors whose metazoan orthologs have not been characterized. Signaling pathway mediators notably included not only Wnt ligands and receptors but also many potential modulators of Wnt signaling including members of three families of secreted antagonists: Dkk (Dickkopf family), sFRP-A (a secreted frizzled-related protein) and Dan1(Cerberus/Dan family of Wnt and TGFβ antagonists) [52]. We also identified secreted proteins that modify the extracellular environment, potentially capable of modulating ligand-receptor interactions through many pathways including Wnt as well as BMP and Hedgehog. Of particular note in relation to Wnt signaling were three heparan sulfate proteoglycan modifiers: two lipases implicated in glycipan cleavage closely related to Notum, and an endosulfatase related to vertebrate Sulf1/Sulf 2 [53], [54]. The *Clytia* Notum sequences, derived from a hydrozoan-specific gene duplication, were named NotumA and NotumO because of their aboral and oral expression territories (see below). The second main signaling pathway that emerged in this analysis was the Notch pathway, with transcripts in the Wnt3-MO embryo-upregulated list coding for the Notch ligand and for two proteins related to Botch, whose *Drosophila* and mouse counterparts inhibit Notch protein processing [55].

Further signaling pathway components and transcription factors with likely conserved developmental roles featured in an extended list of transcripts differentially expressed in Wnt3-MO embryos with significance at p = 0.01 level (z-score +/−3. 3 cutoff; list of additional sequences in File S3). These included yet more potential Wnt regulators: another Wnt ligand WntX1A [40], the transcription factor TCF, and Naked cuticle [56], [57] were under-expressed, while another sFRP in the same orthology group as sFRP-A (named sFRP-B) and MESD (which interacts with the co-receptor LRP5/6 [58], [59]) were overexpressed. Components of other signaling pathways also figured in this extended list, including a TGFβ pathway ligand and cytoplasmic inhibitor (SMAD 6/7), a putative FGF receptor and a VEGF-related ligand. *Clytia* orthologs of the developmentally important transcription factors Goosecoïd (Gsc), Iroquois (Irx), Hox9/14B [16] and Rfx were also identified.

#### Other conserved metazoan genes

The developmental regulator gene orthologs listed above were known through functional studies in classic experimental model species such as *Drosophila*, mouse and zebrafish. Additional ancient metazoan genes conserved during evolution may also have roles in regulating development that have yet to come to light. As well as genes probably associated with the differentiation of larval cell types such as myophilin, calmodulin and innexin, our analysis provided a number of candidate-conserved developmental regulators, falling into three categories: 1) Known genes with little or no previously known involvement in development, for instance, coding for the amino acid transporter Aat or other solute carriers; 2) Members of large gene families associated with developmental regulation but lacking clear bilaterian orthologs, including putative transcription factors containing helix-loop-helix or zinc finger domains as well as 7-pass transmembrane (7tm) proteins (a large and diverse family of receptors for cytokines, hormones, peptides and other ligands with the potential to evolve developmental cell-cell signaling roles, as has occurred in the Frizzled family). 3) As yet uncharacterized genes that have homologs in bilaterians. This third category includes proteins containing conserved domains identified in the PFAM database such as the Domains of Unknown Function DUF4323 and DUF3504.

#### Cnidarian restricted genes

A considerable proportion of the sequences with complete predicted ORFs (37/126 = 29%) did not have identifiable orthologs among known bilaterian genes or any other non-cnidarian sequences in the NCBI databases (see Methods for details), as defined by a lack of significant similarity by reciprocal BLAST along the length of the sequence. These ‘cnidarian restricted’ sequences included the transcript most strongly under-expressed (highest z-score) in Wnt3-MO embryos, WegO1. In some cases, despite the absence of any identifiable ortholog, recognizable conserved motifs such as SAM or PH domains could be detected within the sequence of these transcripts using domain prediction software, suggesting involvement in mediating protein–protein or protein-membrane interactions. These domains are common to many diverse bilaterian proteins and cannot be taken as indicating homology. Such sequences could have originated through domain recombination or through extreme divergence of surrounding sequences during cnidarian evolution. Six of the novel cnidarian-restricted sequences identified in our study had clear counterparts in the fully sequenced genomes of *Nematostella* and/or *Hydra* but the others (31/37) are unique to *Clytia* amongst available sequences. Genome sequences for a larger range of cnidarian species will be required to assess the degree of taxonomic restriction of these genes. Thirteen possessed predicted 5′ signal peptide sequences indicating that they code for secreted proteins varying in length from 77–526 predicted amino acids (average about 260). These characteristics are compatible with roles as novel signaling ligands, antagonists or extracellular regulators.

### *In situ* hybridization analysis reveals four spatiotemporal expression profile types

**Figure 2:**
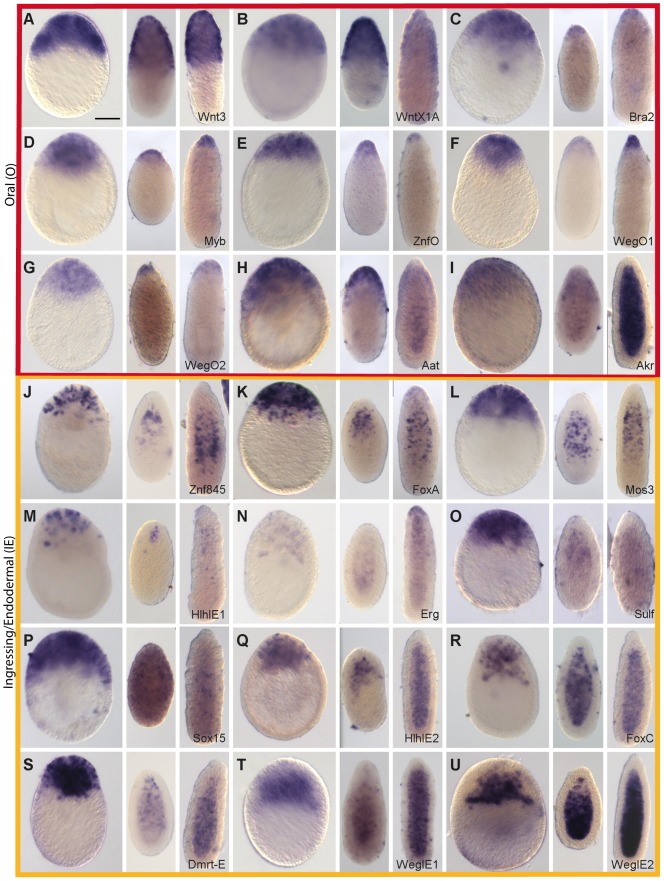
Preferential expression of Wnt3-MO-embryo under-expressed transcripts in oral and ingressing cells. *In situ* hybridization analysis of Wnt3-MO-embryo under-expressed transcripts in early gastrula, 24hpf planula and 48hpf planula stages (from left to right in each panel). The expression of Wnt3 (A; Momose et al., 2008) is compared with that of the 20 down-regulated transcripts with the highest z-scores. All genes showed heavily oral-biased expression at the early gastrula stage, with two principle distribution patterns: Predominantly in the oral ectoderm at all stages (panels A-I; “Oral” expression pattern, outlined in red); Mainly in ingressing/ingressed cells during gastrulation and then in the endodermal region of the planula (panels J-U; ‘Ingressing/Endodermal” expression pattern, outlined in orange). Two of the “Oral pattern” transcripts (panels H and I) also showed expression in the endoderm at the planula stage. Representative images of the patterns observed in at least three experiments are shown. All embryos are oriented with the oral pole uppermost. Bottom right: gene name (see Table 1). Scale bar 50 µm.

**Figure 3:**
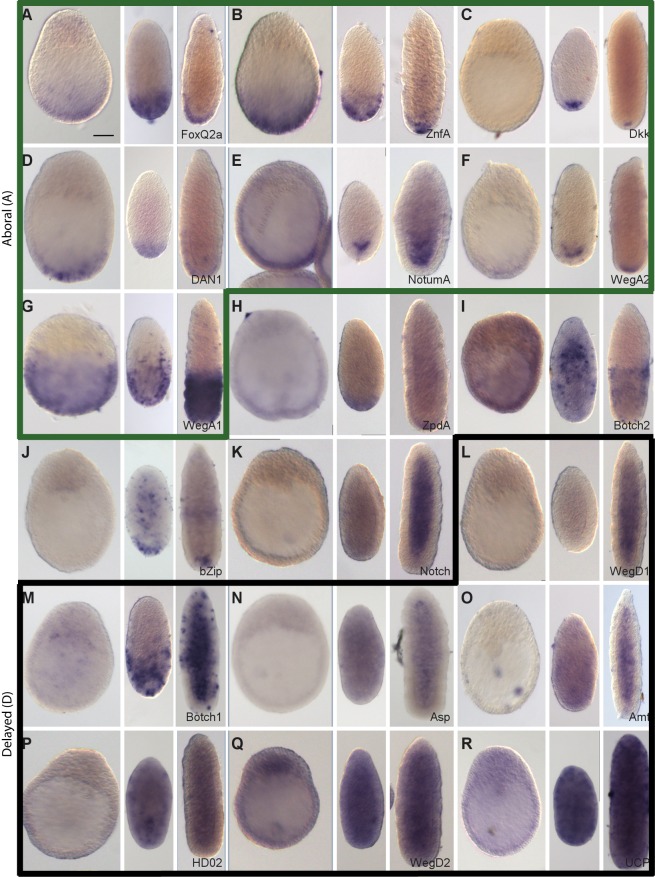
Preferential expression of Wnt3-MO-embryo over-expressed transcripts in aboral domains and planulae. *In situ* hybridization analysis of Wnt3-MO-embryo over-expressed transcripts in early gastrula, 24hpf planula and 48hpf planula stages (from left to right in each panel). The 18 transcripts with the highest z-scores were analyzed. Seven genes showed expression clearly restricted to the aboral territories at planula stages (A-G), already apparent at the early gastrula stage except in in the case of Dkk (C). Two additional transcripts showed an aboral expression pattern but with additional signal in endodermal region of planula larvae: ZpdA (H) and Botch2 (I). 9 transcripts (J-R) showed low ubiquitous or undetectable expression at the gastrula stage followed by a variety of patterns in planula larva including ubiquitous (Q-R), mainly endodermal (K-O) and/or with diverse dynamic distributions of scattered cells in the ectoderm and/or endoderm (J, M, N, O, P). We classified the expression profiles as “Aboral-type” (A-G, panels outlined in green), “Delayed” type (L-R, panels outlined in black) or as showing a mixture of these profiles (4 remaining panels). Representative images from at least three experiments are shown. All embryos are oriented with the oral pole uppermost. Bottom right: gene name (see Table 1). Scale bar 50 µm.

**Figure 4:**
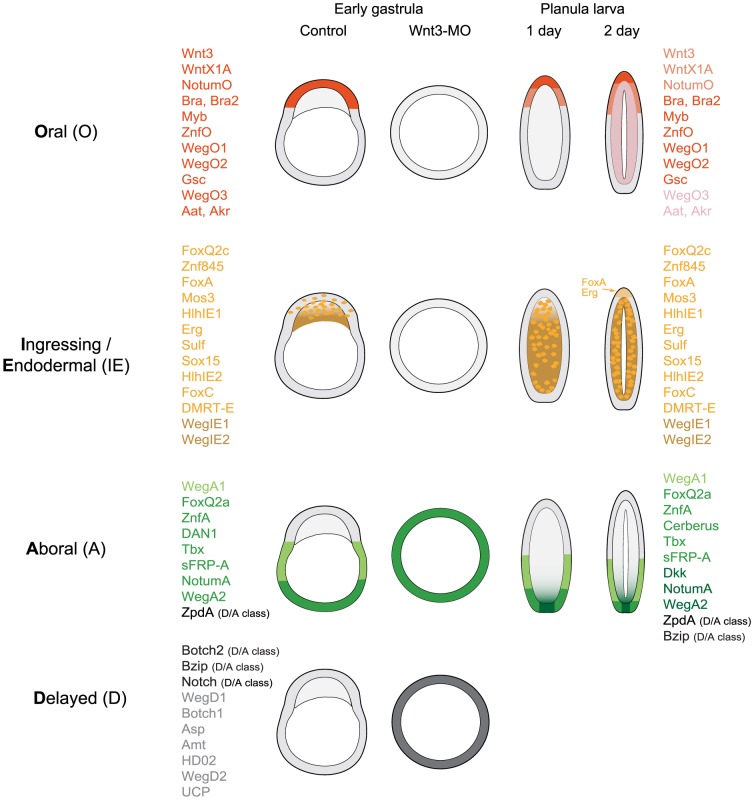
Summary of expression profiles observed for the analyzed transcripts. The differentially expressed transcripts analyzed in this study showed four basic types of expression profile: Oral (oral pole ectoderm at all stages); Ingressing/Endodermal (cells moving into the endodermal region during gastrulation and persisting in this region); Aboral (aboral pole throughout) and Delayed (absent or very low expression at the beginning of gastrulation, then expression in a variety of regions/cell types at the planula stages). Within these four categories there were differences in the limits of detected expression as indicated.

We undertook detailed characterization of spatial expression and sequence analysis (Table 1) non-selectively for the top 20 under-expressed transcripts (Figure 2) and top 18 over-expressed transcripts (Figure 3) in Wnt3-MO early gastrulae. Expression territories for all 38 transcripts were determined by *in situ* hybridization at three stages: early gastrula, 24 hpf planula (just completed gastrulation, endoderm still undifferentiated), and 48 hpf old planula (cell differentiation ongoing in both endodermal and ectodermal regions). We found that almost all the *in situ* hybridization profiles could be assigned to one of four types, which we termed Oral (O), Aboral (A), Ingressing/Endodermal (IE) and Delayed expression (D) types, as described in more detail below and summarized in Figure 4. Briefly, O and A type profiles are characterized by polarized expression with respect to the developing oral-aboral axis at all stages, suggesting ongoing patterning roles during embryonic and larval development. The IE type profile corresponds to cells destined to contribute to the complex endodermal region including the i-cell stem cells and their derivatives. The D type profile transcripts were barely detectable in early gastrulae but showed at larval stages expression in diverse patterns in the ectoderm and/or later in the endoderm. Overall, our approach to identify new candidates for roles in cnidarian embryonic development was completely validated by these analyses. Without any selection based on sequence identity, all the transcripts we tested showed expression restricted in space and/or time during gastrulation and planula development.

**Table 1:** Characteristics of all transcripts analyzed in detail in this study.

Name	Wnt3-MO response (z-score)	Identity/domains	Cnidarian specificity for novel transcripts	Domains	Expression pattern type	DGE class
TRANSCRIPTS UNDER-EXPRESSED IN Wnt3-MO						
WegO1	−24.6	Cnidarian-restricted	*Clytia*		O	1
Znf845	−21.2	Zn-finger domain, expressed in *Hydra* i-cells		DNA binding	IE	2
DMRT-E	−19.1	DM-DNA binding domain. Doublesex and mab-3 related transcription factor		DNA binding	IE	2
HlhIE1	−18.7	HLH domain		DNA binding	IE	1
Mos3	−18.2	Mos kinase			IE	2
Bra2	−18	Brachyury 2 ; T-box transcription factor		DNA binding	O	1
Sulf	−17.5	Extracellular Sulfatase related to Sulf1/Sulf2 (modulates ligand signalling)		Signal peptide	IE	1
FoxA	−16.2	FoxA; Forkhead-domain transcription factor		DNA binding	IE	2
FoxC	−15.5	FoxC; Forkhead-domain transcription factor		DNA binding	IE	2
ZnfO	−14.8	Zn-finger domain		DNA binding	O	2
Sox15	−14.5	Sox domain (Jager et al 2010)		DNA binding	IE	2
WntX1A	−13.8	Wnt ligand, (Momose et al 2008)		Signal peptide	O	1
Akr	−13.6	Small protein with 2 Ankyrin repeats.			O (+ endo)	1
WegIE1	−13.3	Cnidarian-restricted , SAM domain	Hydrozoan		IE	2
HlhIE2	−12.9	HLH domain		DNA binding	IE	2
Aat	−12.4	Amino acid transporter		Trans-membrane	O (+ endo)	1
WegIE2	−12	Cnidarian-restricted, secreted protein	Clytia	Signal peptide	IE	2
WegO2	−11.8	Cnidarian-restricted	Clytia		O	1
Myb	−11.4	Myb-type HTH DNA-binding domain		DNA binding	O	1
Erg	−11.2	Ets DNA-binding domain, SAM/pointed domain		DNA binding	IE	2
*Bra*	*−10.6*	*Brachyury 1; T-box transcription factor (Momose & Houliston, 2007)*		*DNA binding*	*O*	*1*
*FoxQ2c*	*−8.4*	*Forkhead-domain transcription factor*		*DNA binding*	*IE*	*2*
*NotumO*	*−8.4*	*Notum ; extracellular Wnt signalling regulator*		*Signal peptide*	*O*	*1*
*WegO3*	*−5*	*related to human CXorf65 (uncharacterized)*			*O (+ endo)*	*1*
*Gsc*	*−4.6*	*Goosecoid, homeodomain protein*		*DNA binding*	*O*	*1*
	TRANSCRIPTS OVEREXPRESSED IN Wnt3-MO EMBRYOS					
Botch1	18.1	ChaC domain - related to Botch (Notch antagonist)			D	4
bZip	12	bZip TF, related to CCAAT/enhancer-binding proteins		DNA binding	D/A	4
FoxQ2a	11	Forkhead-domain transcription factor (Chevalier et al 2006)		DNA binding	A	3
Dkk	10.2	Dickkopf family (secreted Wnt antagonist)		Signal peptide	A^#^	3
ZnfA	10	Zn-finger domain (SP1 family)		DNA binding	A	3
NotumA	9.3	Notum ; extracellular Wnt signalling regulator		Signal peptide	A	4
Botch2	8.8	ChaC domain - related to Botch (Notch antagonist)			D/A	4
WegD1	8.2	Cnidarian-Restricted - Pleckstrin Homology domain	Hydrozoan		D	4
WegAI	8.3	DUF3504 domain	Cnidarian		A	3
Asp	7.9	Asparaginase, 5 Ankyrin repeats			D	4
WegD2	7.8	Cnidarian-restricted	Clytia		D	4
Dan1	7.3	Dan/Cerberus family of secreted Wnt/BMP inhibitors		Signal peptide	A	3
ZpdA	7.3	Cell Surface Glycoprotein - zona pellucida domain superfamily		Trans-membrane	D/A	4
Amt	6.9	Ammonium transporter		Trans-membrane	D	4
HD02	7	Antp family homeodomain protein (Chiori et al., 2009)		DNA binding	D	4
WegA2	6.9	Cnidarian-restricted , SAM domain	Clytia		A	3
Notch	6.7	Notch		Trans-membrane	D/A	3
UCP	6.8	Mitochondrial Uncoupling Protein			D	4
*Tbx*	*6.5*	*T-box transcription factor*		*DNA binding*	*A*	*3*
*sFRP-A*	*6.2*	*Secreted Fz (sFRP)- lectin domain*		*Signal peptide*	*A*	*3*
*Fz3*	*5.4*	*Fz family receptor (Momose & Houliston, 2007)*		*Signal peptide*	*A*	*3*

Details of transcripts for which *in situ* hybridization analyses were performed previously or in this study, with corresponding expression pattern type (see Figure 4) and DGE class as defined according to z-scores in Wnt3-MO and Fz1-MO transcriptomes compared to non-injected embryos (see Figure 7). Transcripts selected from outside the “top 20” under and over-expressed list are in italics. See text for details. O =  Oral; A =  Aboral; IE =  Ingressing/Endodermal; D =  Delayed. endo =  endoderm in 48hpf planula. #  =  no expression detectable in Early Gastrula.

Names were assigned to the analyzed transcripts on the basis of orthology and/or membership of known gene families (all phylogenetic analyses in File S2). Multiple members of known gene families were distinguished by suffixes designating the 4 main expression profile types: O, A, IE or D. Cnidarian-specific transcripts lacking any recognizable orthologs from non-cnidarian species in NCBI databases, and those with non-cnidarian orthologs that had not previously been characterized, were assigned novel names using the same suffixes, prefixed by “Weg” to denote differential expression in Wnt3-MO early gastrulae, or given names based on recognizable repeats when present.

#### Wnt3-MO embryo under-expressed transcripts show oral and endodermal profiles

Consistent with the aboralized phenotype of the Wnt3-MO-embryos, the twenty top under-expressed transcripts were all strongly localized during normal development to cells at the future oral pole (site of gastrulation) at the early gastrula stage (Figure 2). Their expression profiles could all be designated unambiguously as either O or IE type. The eight O type profile transcripts (Figure 2B-I) were detected strongly in the oral pole ectoderm in both gastrula and planula larva stages. These included the Wnt ligand WntX1A [40], three transcription factors (*Clytia* Bra2, Myb and ZnfO), and two novel cnidarian genes designated WegO1 and WegO2. None of these transcripts were significantly detected in cells ingressing during gastrulation, indicating either expression in exclusively ectodermal cells or down-regulated in ingressing cells upon their separation from the oral ectoderm. Two additional O-type profile genes showed later additional expression in cells of the endodermal region at the planula stage (Aat and Akr; panels H and I). Expression domains for these two genes and for the two Wnt ligands extended across the oral third of the larva [40]. In contrast, expression of the other five genes was predominantly detected at the oral tip, resembling the previously-described expression of Bra1(initially named Bra) [39]. O type expression patterns were also obtained for three additional transcripts selected from the Wnt3-MO-underexpressed list; Gsc, NotumO and an evolutionarily conserved but previously uncharacterized sequence designated WegO3 (*in situ* hybridization images in File S4). Gsc and WegO3 both showed oral tip expression, supplemented with additional endodermal expression in 48 h planula larvae for WegO3. NotumO expression extended further along the oral-aboral axis, matching that of the two Wnt ligands.

IE type patterns were observed for twelve transcripts, and were characterized by expression mainly in ingressing or ingressed cells during gastrulation, and later in different cell populations within the endodermal region (Figure 2J-U). At the gastrula stage, these transcripts were detected in subpopulations of cells ingressing into the endoderm at the oral pole, as well as in some cases putative pre-ingressing populations in the ectoderm. At planula stages, they were detected predominantly in the endodermal region, in different sub-populations of cells. For the transcription factors Znf845, FoxA and also for the kinase Mos3, the distribution of expressing cells, notably their position in non-polar regions between the endoderm and ectoderm layers in 48 hpf larvae, was reminiscent of previously described germ line/stem cell genes expressed in i-cells such as Nanos1, Vasa and Piwi [60]. Expression in scattered ingressing cells was also observed for HlhIE1 (Figure 2M; fewer cells detected), the Ets family transcription factor Erg (Figure 2N; additional expression in oral ectoderm cells in 48 h planulae), *Clytia* Sulf (Fig 2O; very weak in 48hpf larvae) and Sox15 (Figure 2P; additional expression in various endodermal and ectodermal cells as previously described [17]), and also for an additional FoxQ2 paralog identified in the Wnt3-MO-underexpressed transcript set designated FoxQ2c (File S2). The hypothesis that Znf845 and FoxQ2c were expressed in i-cells or their primary derivatives is consistent with data from a recent study in *Hydra* which compared transcriptomes of sorted endodermal, ectodermal or Nanos-expressing (i-cell lineage) cells from adult polyps [25] (see File S5). HlhIE2, DMRT-E and FoxC were also expressed in early stages of ingression in the early gastrula but adopted more widespread distribution through the endodermal region in the planula. Correspondingly *Hydra* FoxC transcripts are highly enriched in endodermal cells [25] (File S5). Two novel cnidarian transcripts (WegIE1 and WegIE2) were detected predominantly in an extensive population of presumptive endoderm cells, expression only becoming detectable once they have entirely separated from the oral ectoderm (Figure 2T,U). Additional WegIE1 expression in scattered larval ectoderm cell populations was observed, particularly in 24 h planulae (Figure 2T).

#### Wnt3-MO embryo over-expressed transcripts are detected in aboral territories or repressed in early gastrulae

Spatial expression profiles obtained for the top eighteen Wnt3-MO embryo up-regulated genes (Figure 3) were of the A (Aboral; Figure 3A-G) or D (Delayed; Figure 3L-R) profile types, or in four cases showed characteristics of both profile types (Figure 3H-K). The seven transcripts assigned as having clear A-type profiles, on the basis of sustained expression at the aboral pole, notably included the Wnt regulators Dkk1/2/4, Dan1 and NotumA, the transcription factors FoxQ2a and ZnfA, the novel cnidarian gene WegA2, and WegA, which has no clear non-cnidarian orthologs but contains a conserved 135aa domain (DUF3504). Six of these seven transcripts were detectable from the gastrula stage, while Dkk1/2/4 was only detectable in planulae. The extent of the aboral expression territory varied between genes, with WegA1 expression extending along about half the oral-aboral axis at all stages, while the others were expressed in the aboral third at the early gastrula stage before becoming more tightly restricted to different ectodermal and/or endodermal cell populations at the aboral pole of the planula larva. Consistent with the ectodermal localization of this transcript, a WegA1 ortholog identifiable in published transcriptome data from *Hydra* polyps [25] is also preferentially expressed in ectoderm (File S5). Clear A-type expression profiles were also observed for two additional transcripts selected on the basis of sequence identity from lower down the Wnt3-MO-underexpressed list: Tbx and sFRP-B (File S4).

Four more transcripts from the “top 18” showed aborally enhanced expression in at least two of the three stages tested, but also additional expression at other sites. Given their barely-detectable expression at the early gastrula stage they had partial expression features of both the A and D type profiles (see below). The extracellular glycoprotein ZpdA showed enhanced aboral expression at the gastrula and 24 h planula stages, but was detected across the whole larva by 48hpf (Figure 3H). Conversely, expression of Botch2 was mainly confined to cells in the aboral half ectoderm at 48hpf, but concentrated in the oral endoderm at 24hpf (Figure 3I), while bZip-expressing cells were concentrated at the aboral pole at planula stages but also detected in more central locations (Figure 3J). Notch expression was not detectable in the gastrula and was predominantly endodermal in planula stages, but with low additional signal detected in the aboral ectoderm (Figure 3K).

The seven transcripts with D type profiles exhibited a heterogeneous array of expression sites (Figure 3L-R). Their main common characteristic was ubiquitous low or undetectable expression at the gastrula stage, later being detected in a variety of distributions. WegD1 transcripts were detected only in the endodermal region at 48hpf (Figure 3L); Botch1 transcripts were detected initially in cells of the aboral ectodermal in 24hpf planulae but at 48h in patches of cells scattered irregularly along the oral-aboral ectoderm as well as through the endodermal region (Figure 3M). The Asparaginase (Asp; Figure 3N) and Ammonium transporter (Amt; Figure 3O) transcripts could be widely detected in cells in both cell layers in 24hpf planulae and then mainly in individual cells of the endodermal layer one day later. Finally the homeodomain protein HD02 (Figure 3P), WegD2 (Figure 3Q) and the mitochondrial uncoupling protein UCP (Figure 3R) were detected in cells distributed widely across the larva at both 24 and 48hpf.

**Figure 5:**
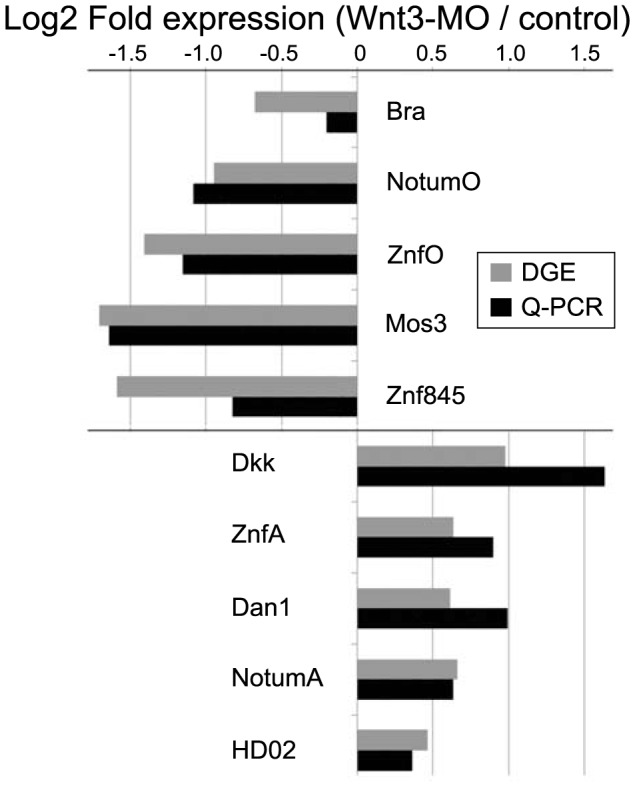
Equivalent differential expression responses determined by DGE and Q-PCR. For ten selected transcripts from different DGE classes and with different expression profiles (see Table 1, Figure 4), transcript levels at the early gastrula stage were determined by Q-PCR in Wnt3-MO and non-injected early gastrula embryos. The ratio of expression levels of selected genes between injected and control embryos, normalized with respect to EF-1α is compared to the DGE data represented in the same way by using the counts of reads mapped rather than the number of cycles of Q-PCR amplification. Transcript identities are shown beside each pair of bars.

**Figure 6:**
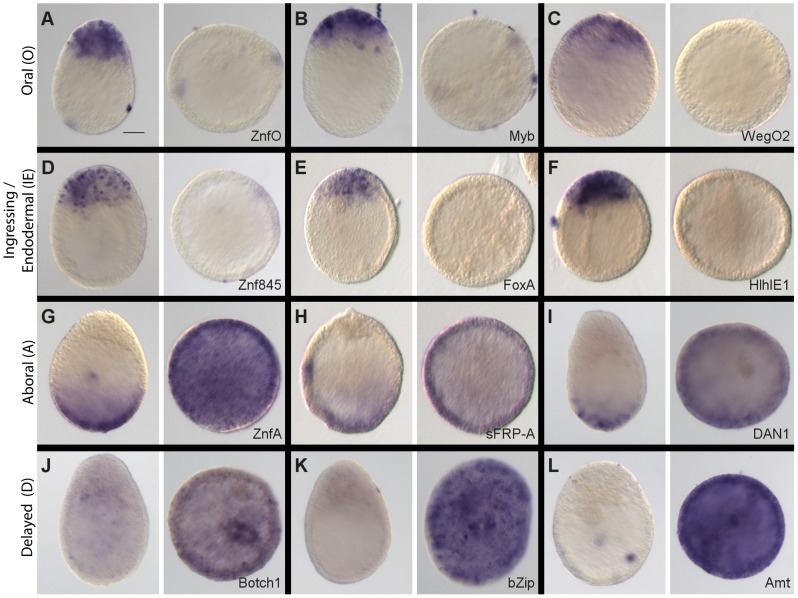
Stereotyped modification of gene expression patterns in Wnt3-MO embryos. *In situ* hybridization analysis of selected transcripts in untreated (left panels) and Wnt3-MO injected (right panels) early gastrulae. Each expression pattern type is represented in the transcripts analyzed, as indicated on the left. A-C: O-type pattern transcripts; D-F: IE-type pattern transcripts, G-I: A-type pattern transcripts; J-L: D-type pattern transcript. For Botch1 (J) and bZip (K), individual cells with high expression on the blastocoelar face of the ectoderm were discernible in the Wnt3-MO embryos. Representative images of the patterns observed in at least three experiments are shown. All control embryos are oriented with the oral pole uppermost. Gene identity is shown in the bottom right of each pair of panels. Scale bar 50 µm.

Unlike the regionalized A, O and IE type patterns, the diverse expression profiles of this D group of transcripts was not anticipated from the aboralized Wnt3-MO phenotype. To check that they did not represent false positives from the DGE screen, we verified expression for representatives of each of the four expression profile types in Wnt3-MO embryos by quantitative PCR (Q-PCR; Figure 5) and *in situ* hybridization (Figure 6). The Q-PCR analysis confirmed the DGE response for all of the10 transcripts tested. By *in situ* hybridization, expression of O and IE profile transcripts was as expected undetectable in Wnt3-MO early gastrula. D-type pattern transcripts showed strongly elevated expression in Wnt3-MO embryos compared to control embryos processed in parallel. The expressing cells lined the blastocoel across the embryo for Botch1, bZip and Amt (Fig 6. J-L); also found for Asp, ZpdA, and Botch2 (File S6). In contrast, A type profile transcripts (Fig 6. G-I) showed expression territories extended spatially though the ectoderm from the aboral side, but without significantly higher expression than in the aboral domain of control embryos. These results indicate that IE, A and O type profile genes are all regulated by regional differences in Wnt signaling activity at the early gastrula stage, whereas D type profile gene transcription is activated temporally between the early gastrula and planula stages following down-regulation of Wnt3 signaling.

### PCP disruption preferentially affects transcripts with non-axial expression profiles

**Figure 7:**
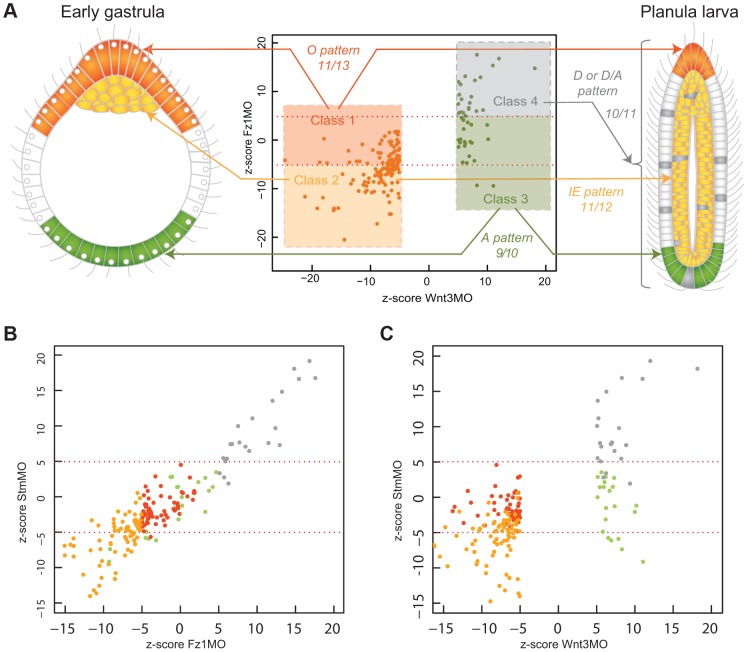
DGE responses correlate with spatial expression profiles. A) Schematic representation of the 4 types of expression profile observed amongst characterized transcripts (see Figure 4) at early gastrula (left) and planula (right) stages, showing their mapping onto the differential responses of the transcripts to Wnt3-MO and Fz1-MO. indicated on the z-score plot in the center. Four DGE classes were defined on the basis of z-scores in Wnt3-MO and Fz1-MO embryos applying cutoffs of -5 for classes 1 and 2, and +5 for classes 3 and 4 as indicated respectively by the dark and light orange, green and gray zones on the graph. The numbers indicate how many of the transcripts for which expression patterns were determined for each class showed the corresponding expression profile. These transcripts are all listed in Table 1 and with expression patterns shown in Figures 2, 3 and 6. B) Equivalent Z-score plot mapping a transcriptome dataset for Stbm-MO early gastrula-stage embryos against the Fz1-MO dataset. Dot colors correspond to the 4 classes defined in A. There is a strong correlation between expression responses in these two conditions, especially for classes 3 and 4 (green and grey dots, ie transcripts over-expressed in Wnt3-MO embryos). C) Z-score plot mapping the Stbm-MO transcriptome dataset embryos against the Wnt3-MO dataset. This illustrates that the 4 DGE classes defined on the basis of Fz1-Mo and Wnt3-MO responses situate largely but not exclusively in the equivalent zones of the Stbm-MO vs Wnt3-Mo graph.

The overall outcome of our *in situ* hybridization analyses was that transcripts identified as Wnt3-MO-underexpressed consistently showed Oral and Ingressing/Endodermal type expression profiles while the overexpressed ones all showed Aboral and Delayed type profiles. The significance level of the response did not, however, correlate with expression patterns (O versus IE or A versus D, respectively; see z-scores in Table 1). Remarkably, we were able in both cases to uncover a strong correlation when we included in the analysis the z-scores obtained for the Fz1-MO sample (Figure 7A). This could be demonstrated by plotting the z-scores calculated for the two experimental conditions (against non-injected) against each other and determining the position of all the transcripts analyzed in Figures 2 and 3, of genes with expression patterns characterized previously (Bra, Fz3) and of five additional examples selected from our primary list (FoxQ2c, Tbx; NotumO, sFRP-A, Gsc, WegO3; File S4; All patterns summarized in Table 1 and Figure 4). Amongst the Wnt3-MO embryo under-expressed transcripts (orange dots in Figure 7A), those with Fz1-MO z-values higher than -5.0, ie not significantly affected or only relatively weakly underexpressed in Fz1-MO embryos, tended to show the O type expression pattern (eleven of the thirteen examined transcripts in the dark orange “Class 1” zone). The others (pale orange “Class 2” zone) showed IE type expression profiles in eleven of the twelve cases. A similar strong correlation was found for the Wnt3-MO embryo-over-expressed transcripts (green dots in Figure 7). In this case, applying a Fz1-MO z-score value threshold of +5.0 we found that transcripts with higher z-values (grey “Class 4” zone) tended to show D or mixed D/A-type patterns (seven and three respectively of the eleven analyzed transcripts), while nine transcripts with z-scores less than 5.0 (green “Class 3 “zone) showed A-type patterns and the tenth (Notch) a mixed A/D pattern. In this Class3 zone, responses to Fz1-MO were quite variable, including moderate over-expression, unchanged expression and, in a few cases, under-expression (notably FoxQ2a and WegA1).

From these analyses we defined four “DGE classes” on the basis of z-score values in Wnt3-MO and Fz1 MO embryos, as indicated in Figure 7A. Although these classes strongly correlate with the four types of expression profiles (Figure 7; Table 1) there are exceptions, for instance ZnfO is categorized as Class 2 on the basis of z-scores but shows an oral type expression profile, while Sulf is categorized as Class 1 but shows endodermal expression.

**Figure 8:**
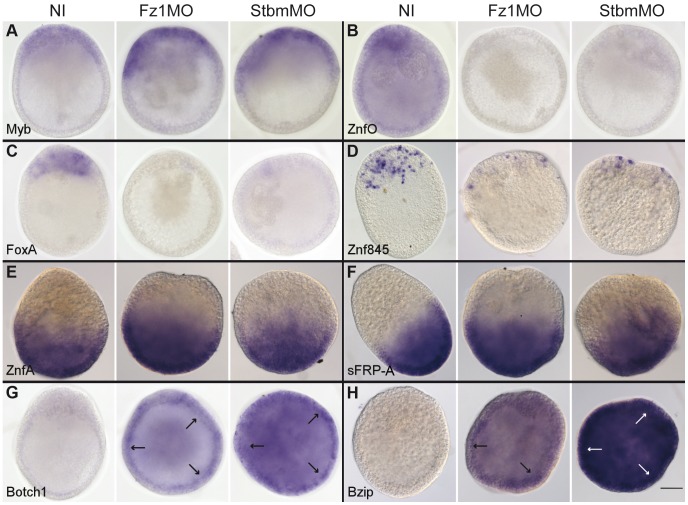
Fz1-MO and Stbm-MO have very similar effects on gene expression profiles. Each set of 3 panels shows typical *in situ* hybridization patterns obtained for uninjected embryos (left), Fz1-MO embryos (center) and Stbm-MO embryos (right) injected, fixed at the early gastrula stage and processed in parallel. A-B: O-type pattern transcripts (Myb and ZnfO); C-D: IE-type pattern transcripts (FoxA and Znf845); E-F: A-type pattern transcripts (ZnfA and sFRP-A); G-H: D-type pattern transcripts (Botch1 and bZip). Cells with high Botch1 and bZip expression on the blastocoelar face of the ectoderm were discernible (narrows) in Fz1MO and StbmMO embryos. Gene identity is shown in the bottom left of each set of panels. For each transcript the two morpholinos have very similar effects on the expression patterns, confirming the z-score comparisons (File S1). Scale bar 50 µm.

Fz1 acts as a receptor for Wnt3 to activate Wnt/β-catenin signaling [39], [40], but is also thought to interact with the *Clytia* Strabismus protein to mediate planar cell polarity (PCP), necessary for cell alignment in the ectoderm but also axial elongation during larval development and endoderm formation [41]. We thus hypothesized that the differences in expression responses in Fz1-MO versus Wnt3-MO could be due to the specific involvement of Fz1 in PCP. To test this hypothesis we made additional comparisons using a transcriptome derived from early gastrula embryos in which PCP was specifically disrupted by a morpholino targeting Strabismus (Stbm-MO). Plotting the z-scores (in relation to uninjected embryos) of the Fz1-MO and Stbm-MO transcriptomes against each other revealed a striking similarity (Figure 7B). The linear positive correlation was especially clear between Fz1-MO and Stbm-MO z-scores for the Wnt3-MO over-expressed transcripts (i.e. DGE Classes 3 and 4; green and grey dots respectively in Figure 7B; Pearson correlation coefficient value 0.93). The separation between Class 1 and Class 2 transcripts on the basis of Stbm-MO responses was less strict, with Class 1 transcripts showing moderately increased or decreased levels in these embryos, compared to unaffected or reduced levels in Fz1-MO embryos (compare distribution of orange dots in Figure 7A and 7C). This can be explained by the requirement of Fz1 but not Stbm in Wnt/β-catenin signaling in the presumptive oral territory,

We validated the transcriptome comparison analyses by *in situ* hybridization on Fz1-MO and Stbm-MO early gastrula embryos (Figure 8) using a subset of the probes used to examine Wnt3-MO embryos (Figure 5). For each gene the expression patterns in the two morpholino conditions were strikingly similar: The Class 1/O-type pattern transcript Myb, and the Class 3/A-type pattern transcripts ZnfA and sFRP-A showed little change compared with non-injected controls (Figure 8A, E, F). ZnfO, assigned to DGE Class2 despite its O-type expression profile, showed undetectable expression at the early gastrula stage in both Fz1-MO and Stbm-MO embryos (Figure 8B) and thus indeed represents an axially-expressed gene atypically sensitive to PCP perturbation.

The weak change in levels of most axially-expressed genes along with the significant under-expression of FoxQ2a and WegA1 in both Fz1-MO and Stbm-MO early gastrula embryos (Figure 7A, C; File S1) revealed in this study may at first seem difficult to reconcile with the previous description of an “aboralized” phenotype including a slight expansion of the FoxQ2a expression domain in Fz1-MO embryos [39], but this can be explained by a difference in the timing of the two studies since the PCP effect is only transient. Thus, analysis of Stbm-MO embryos revealed that while aboral FoxQ2a expression is undetectable by *in situ* hybridization at the early gastrula stage it subsequently becomes restored, while conversely oral expression of Bra1 is transiently expanded but then becomes re-restricted to the oral pole of the planula [41].

The *in situ* analyses performed for Class 2/IE-type and Class4/D-type pattern transcripts also validated the DGE analyses. FoxA and Znf845 were barely detectable by *in situ* hybridization at the early gastrula stage (Figure 8C, D), while Botch1 and bZip were detected strongly across the embryo (Figure 8G, H). As in Wnt3-MO embryos (Figure 5) the signal in these latter cases was mainly detected in cells positioned on the basal side of the ectodermal epithelial layer.

We conclude that the relatively strong under-expression (Class 2) or over-expression (Class 4) of certain genes in Fz1-MO embryos is due in whole or part to disruption of PCP. This effect could reflect regulation of gene transcription by specific signaling pathways activated by PCP or be indirect, resulting from disturbed morphogenesis following failure of the ectodermal cells to align, to develop cell polarity and to undergo ciliogenesis [41].

### Knockdown of conserved and cnidarian-restricted genes generates developmental defects

**Figure 9:**
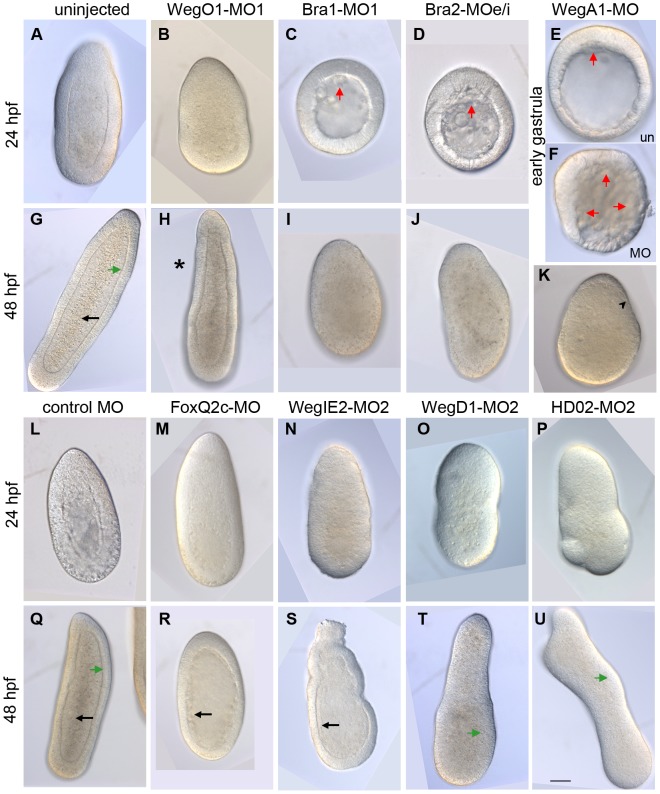
Morpholinos targeting conserved and cnidarian-specific transcripts disrupt development. DIC imaged larvae developed from morpholino-injected eggs after 24hpf and 48hpf development or at the early gastrula stage as indicated. Typical morphology observed at non-toxic morpholino doses are shown in each case, with the oral pole at the top. Similar phenotypes were obtained using a second morpholino in all cases, except FoxQ2c and WegA1, for which no second non-toxic morpholino could be designed. Uninjected embryos (A) and control-MO injected embryos (L) had completed gastrulation at 24hpf with endodermal epithelialisation starting at the aboral pole. By 48hpf uninjected planula larvae (G, Q) had formed with endodermal epithelial layers (black arrows) organized around a central stripe-like cavity and a well-defined lamina layer separating the endoderm and ectoderm (green arrows). WegO1-MO1 embryos showed minor disruption of gastrulation with embryo elongation slightly compromised at 24hpf (B) and the oral half, thin and tapered (asterisk) at 48hpf (H). Morpholinos targeting either Bra1(C, I) or Bra2 (D, J) showed severe delays in gastrulation. By 48 hpf some embryo elongation had occurred but the blastocoel contained only loose, disorganized material. WegA1-MO embryos (F) showed massive cell ingression at the onset of gastrulation, when cell ingression had barely initiated in uninjected embryos cultured in parallel (E). At 48 hpf (K) the endoderm was enlarged and the ectoderm layer was very thin and irregular (black arrowhead). FoxQ2c-MO (M, R) and WegIE2-MO (N, S), showed severely reduced endodermal layer at 48hpf. WegD1-MO and HD02-MO embryos showed severe defects in overall morphology (O, P, T, U), lacking a well defined basal lamina between endoderm and ectoderm. Green arrows indicate the position of this interface. Black arrows indicate the endodermal epithelial layers in G, Q, R and S. Red arrows indicate ingressing cells at the onset of gastrulation in C, D, E and F. Scale bar 50 µm for all panels.

To test whether the newly identified genes in *Clytia* were indeed involved with developmental processes as predicted by their expression patterns, we injected antisense morpholino oligonucleotides targeting a selection of identified genes. We included in this analysis transcripts representing each of the four expression profile types including cnidarian-restricted genes (WegO1, WegIE2, WegD1), candidate conserved developmental regulators (Bra1, Bra2, FoxQ2c, FoxQ2a, HD02) and the partly conserved transcript WegA1. For each morpholino tested, developmental defects observed at morphological (Figure 9) and cellular (File S8) levels were coherent with the corresponding expression patterns (Figures 2 and 3), confirming the usefulness of our approach to identify developmental regulators. Wherever possible (6/8 cases, see File S7 for details) morpholinos targeting two different sites in the transcript were used, and in each case similar phenotypes were observed.

Morpholinos to the three O-type expression pattern transcripts all showed defects in endoderm formation, consistent with endoderm fate specification in the oral territory [61], [62]. Morpholinos targeting the two *Clytia* paralogs Bra1 and Bra2 both significantly inhibited endoderm formation. Initial signs of cell ingression at the oral pole occurred with only a slight delay with respect to non-injected controls, but subsequent filling of the blastocoel was strongly retarded, such that by 24hpf Bra1-MO and Bra2-MO embryos (Figure 9C, D) resembled uninjected embryos at the onset of gastrulation (about 11hpf). Bra1-MO and Bra2-MO embryos then elongated somewhat and disorganized cells accumulated in the blastocoel to a variable degree, although often with a significant reduction in the amount of endoderm observed. Confocal microscopy confirmed that the residual ectodermal cells of both Bra1-MO1 and Bra2-MOe/i embryos accumulated in aboral regions and showed signs of epithelialization (File S8 C, D). A similar but much less severe delay in gastrulation was obtained following injection of morpholinos targeting the cnidarian-restricted gene WegO1, whose expression profile is very similar to that of Bra1 and Bra2 (Figure 2). Planulae showed a characteristic tapering of the oral half (Figure 9H), and confocal microscopy revealed that endoderm was reduced in this region (File S8 B).

Strikingly, morpholinos targeting the A-type profile transcript WegA1 generated an opposite phenotype from the O-type pattern morpholinos. At the onset of gastrulation, massive cell ingression initiated widely across the embryo (Figure 9F). This is reminiscent of the phenotype previously described for Fz3 MO [39]. During subsequent development, cells from the internal regions were expulsed in most embryos, so that by the planula stage, embryos were commonly smaller and consisted of accumulations of endodermal-type cells surrounded in some cases by a very thin ectoderm layer, in which the cells were stretched over the inner cell mass (Figure 9F; File S8 G, H, I).

Morpholinos targeting the two IE type pattern genes WegIE2 and FoxQ2c both caused only minor disruption of development prior to the end of gastrulation, but subsequent formation of the endodermal cell layer was affected, with in both cases a thin and uneven layer of endodermal cells observed at 48hpf surrounding a distended cavity containing cell debris (Figure 9R, S). WegIE2-MO embryos showed additional disorganization of the oral ectoderm. Confocal microscopy confirmed that the endodermal cell layers were severely disorganized (File S8 E,F).

Finally, morpholinos targeting the two D-type profile genes, which are strongly up-regulated at the early gastrula stage upon Wnt3, Fz1 or Stbm disruption, did not markedly disrupt gastrulation but resulted in highly aberrant morphology of the planulae (Figure 9T,U). WegD1-MO embryos showed a distended aboral end with the ectoderm then becoming highly folded, this effect extending along the length of the embryo in the most extreme cases. Injection of morpholinos targeting the ANTP family gene HD02 also resulted in elongated and very irregular shaped planulae. In both cases the interface between the ectoderm and endoderm layers was very irregular with confocal microscopy revealing mixing of cells from the two layers and an absent or highly disrupted basal lamina between them (File S8 P, T). In HD02-MO embryos, anti-tubulin staining revealed an abundance of neurite–like projections traversing irregularly this interface, contrasting with the well defined epithelial basal lamina and regular distribution of orthogonally extending neural projections in undisturbed planulae (File S8; compare K and O).

### Preferential association of cnidarian-restricted genes with embryo patterning

**Figure 10:**
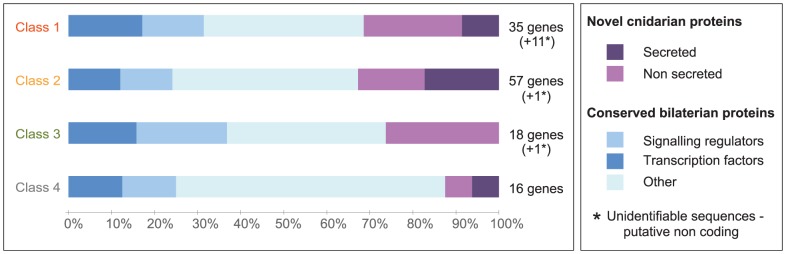
Conserved and cnidarian-specific genes in all DGE classes. Analysis performed on a sub-set of 126 transcript sequences in which predicted ORFs were complete at both 3′ and 5′ ends. Proportions of novel and conserved gene types, including transcription factors and signaling pathway regulators are similar in all groups except DGE class 4, which includes fewer cnidarian-specific genes.

We used the strong correlation between DGE classes and expression patterns to assess the relationship between transcript identity and localization, using the 128 transcripts for which complete ORFs were present (Figure 10). The proportions of transcription factors and probable signaling pathway regulators were similar between DGE classes (12–21%; values not significantly different by Fisher's Exact Test). In contrast there was a significantly higher proportion of cnidarian-restricted sequences in DGE classes 1, 2 and 3 than in DGE class 4 which tend to show D-type expression profiles (around 30% vs 6%; Fisher's Exact Test p-value for this comparison  = 0.04). This analysis suggests that while cnidarian-restricted developmental regulators contribute significantly to patterning at the early gastrula stage, expression of evolutionary ancient genes predominates during development of the larva following gastrulation.

## Discussion

This study successfully identified many potential developmental regulators from the cnidarian experimental model *Clytia hemisphaerica* by analyzing the transcriptome of early gastrula stage embryos aboralized by Wnt3 knockdown, providing a number of new insights into the evolution of developmental patterning mechanisms. Firstly, the key role of Wnt signaling in embryo patterning was confirmed since the identified genes all displayed one of four basic expression profiles, three associated with embryo patterning (through localized expression in the oral, aboral and presumptive endoderm regions) and one with planula formation. Expression profile types could be related to differential expression sensitivity to Wnt3-MO vs Fz1-MO or Stbm-MO, allowing us to separate genes expressed along the oral–aboral axis predominantly under Wnt/β-catenin signaling regulation from genes whose expression at the early gastrula stage is affected by Fz-PCP. Secondly, the identified genes included not only members of known conserved metazoan developmental gene families, but also previously uncharacterized or understudied conserved metazoan genes, providing novel candidates for evolutionary ancient roles in directing developmental processes. Finally, a number of cnidarian-restricted genes emerged as potential developmental regulators. Roles in larval patterning and morphogenesis were confirmed by morpholino analysis for 3 such genes as well as for one that shares a domain of unknown function with bilaterians. Overall our study illustrates the power of systematic transcriptomics-based screens, coupled with functional studies, to identify developmental genes in non-bilaterians and thus to help understand metazoan evolution and diversification.

### Wnt signaling and PCP direct gene expression programs in the early gastrula

Our findings confirmed the central importance of Wnt signaling in embryo patterning. The transcripts under–represented in the spherical, aboralized Wnt3-MO embryos were during normal development systematically found expressed either in the oral ectoderm or in cells that contribute to the endodermal region (defining O and IE type profiles respectively), while those from the over–represented set were detected either in the aboral ectoderm (A type profile) or generally repressed throughout the embryo at the early gastrula stage to be expressed in different patterns during planula larva formation (D type profile). The O and A type profile genes displayed sustained localized expression at the poles through gastrulation and larval development and are thus good candidates for roles in patterning along the oral-aboral axis, but may also include precociously expressed gene markers of larval cell types enriched at one pole.

We were intrigued to find that the four types of expression profile for Wnt3-MO-differentially expressed transcripts strongly correlated with four “DGE classes”, distinguished by the strength of the effect of Fz1-MO on the expression of the same genes. More specifically the axially expressed transcripts tended to show less extreme changes in expression in Fz1-MO early gastrulae than did IE and D-type profile transcripts (Figure 7A). We have shown previously that Wnt/β-catenin signaling activated by Wnt3 and Fz1 is a key regulator of gene expression along the oral-aboral axis [39], [40]. The relatively weak difference in expression of the axial genes in Fz1-MO relative to Wnt3-MO early gastrulae documented here could be explained, at least in part, by incomplete inhibition of this pathway by Fz1-MO compared with total extinction by Wnt3-MO, as revealed by β-catenin nuclear localization (compare Figures 3 in [39] and [40]). It is also conceivable that Wnt receptors other than Frizzleds such as RYK or ROR2 [63] could be partly responsible for mediating the Wnt3 responses in oral regions. Our Stbm-MO analyses demonstrate, however, that the main explanation for less marked changes in expression of ‘axial’ versus ‘non-axial’ genes in Fz1-MO embryos relates to the involvement of Fz1 in PCP. One aspect of this is that transient up-regulation of some oral genes and down-regulation for some aboral genes due to PCP disruption, as shown in Stbm-MO embryos[41], could in Fz-MO embryos counterbalance and dampen the effects of Wnt/β-catenin signaling. Concerning the non-axial genes the strong effects of PCP disruption could reflect direct signaling through ‘non-canonical’ intracellular pathways acting downstream of Fz/Dsh [64]. Given the transient nature of the effect, however, we favor the possibility that the effect is indirect, resulting from the developmental programs of the corresponding cell lineages being delayed or accelerated by a changed morphological environment. For cells of the presumptive endodermal region (IE type pattern), lack of detection at the early gastrula stage in Fz1-MO and Stbm-MO embryos could result from disruption of ingression behavior due to loss of polarity of oral ectoderm cells. Conversely the strong over-expression of the D-type profile genes at the early gastrula stage in Fz1-MO and Stbm-MO embryos suggests that epithelial PCP may have a significant effect in delaying the development of certain planula cell types. One attractive possibility is that Fz-PCP disruption affects apical-basal polarity of epithelial cells and thus the generation of new cell types through oriented asymmetric divisions, as has been recently demonstrated in *Xenopus* embryos [65]. Consistent with this hypothesis, cells expressing Botch1, bZip and Amt became prominent in basal regions of the epithelial ectoderm of early gastrulae when PCP was disrupted directly using Stbm-MO or Fz1-MO (Figure 8), or disturbed indirectly in the Wnt3-MO context [41](Figure 6). Furthermore several other D type profile transcripts (HD02, UNC, WegD2 and possibly also Notch and Botch2) also tended to be expressed in basal regions of the ectodermal and/or endodermal epithelia during planula development (Figure 3).

### Conserved metazoan developmental regulators in cnidarian embryogenesis

Our study provides further support for the well-known idea that a common set of transcription factors diversified from a common cnidarian-bilaterian ancestor has retained roles in regulating development in individual evolutionary lineages, with some families diversifying functions following lineage-specific gene duplications[4]–[6], [9]. *Clytia* orthologs of many of known developmental regulator genes were identified from our unbiased screen based on sensitivity to Wnt/Fz signaling. All those tested showed characteristic spatiotemporally restricted expression profiles, and for four examples from well-known transcription factor families, roles in developmental regulation were supported by functional studies based on morpholino injection. Analysis of the morphant phenotypes suggested that the two *Clytia Brachury* paralogs Bra1 and Bra2, expressed at the oral pole throughout larval development, both play important roles in controlling the progression of gastrulation. Expression around the blastopore has been proposed to be an ancestral metazoan characteristic of *Brachury*, which during bilaterian evolution became involved in the specification of various mesoderm and endoderm fates from these tissues [66] but with the ancestral role likely to have been in regulating morphogenetic movements [67]. In *Clytia*, although there is no blastopore, the relationship with the gastrulation initiation site is conserved, and our morpholino results suggest that morphogenetic movements upstream of endoderm specification are affected. The *Hydra Bra1* and *Bra2* orthologs have been shown to have subtly distinct roles in endoderm and ectoderm layers of the budding polyp [11], suggesting that while embryogenesis roles for these genes overlap, their functions at other life cycle stages have diverged. A morpholino targeting FoxQ2c, expressed in the developing endodermal region during planula formation, caused severe defects in the organization of the endodermal layer. As with *Brachyury*, gene duplications have expanded the *FoxQ2* gene family in Cnidaria, and in this case the paralogs have adopted clearly distinct expression profiles, FoxQ2a having conserved the likely ancestral aboral (anti-blastoporal) expression [68] while FoxQ2b is only expressed in oocytes [15]. The final member of a known developmental transcriptional regulator gene family we tested functionally was HD02, a non-Hox member of the Antp homeodomain family [16], expressed particularly strongly in cells at the base of the ectoderm and endoderm layers during larval development (Figure 2P). The phenotypes following morpholino injections suggest that HD02 is involved directly or indirectly in regulating development of the neural network that develops at this site [69], perhaps dependent on the correct organization of the basal lamina. Further in depth studies will be required to explore this possibility, as well as to confirm and understand fully all the other morpholino phenotypes documented here.

On the basis of expression patterns it is likely that several other transcription factor genes identified in this study have developmental functions conserved through metazoan evolution. For example FoxA and FoxC are associated with distinct cell populations contributing to the endoderm region during gastrulation, as has also been reported for their *Nematostella* orthologs expressed in distinct regions of the developing pharynx [12], [70]. In bilaterian species orthologs of these Fox genes are associated with development of endoderm/axial mesoderm and mesoderm respectively [71]–[74].

As well as transcription factors from families such as T-box, Fox and Antp, our transcriptome comparison identified likely regulators of a variety of intercellular signaling pathways including Notch, FGF, TGFβ and Ras-MAPkinase. These included core components (ligands, receptors and secreted antagonists), but also less well known regulators acting in ligand or receptor processing and/or extracellular interactions, such as the Botch, Sulf and Notum proteins. Most strikingly we identified *Clytia* orthologs of known Wnt pathway regulators acting at all levels: Wnt ligands (WntX1A), receptors (Fz3, Fz2), members of three of the five families of secreted antagonists known from bilaterian models (Dkk1/2/4; Dan1; two sFRPs) [52], [75], MESD which specifically interferes with ligand co-receptor LRP5/6 [58], [59], an ortholog of the intracellular negative regulator Naked Cuticle [56], and also the two Notum family lipases and Sulf. Sulf enzymes act on cell surface Heparan Sulphate Proteoglycans and have been reported to modulate Wnt as well as Hedgehog, TGFβ and FGF signaling while Notum releases the GPI anchor of glycipans such as Dally [76]–[79]. The oral expression profile of all the positive Wnt pathway regulators from this and our previous study (five Wnt ligands, Axin and TCF) reinforces the notion that an active Wnt signaling source is maintained at the cnidarian embryo and larval oral pole [40], [42], [80] as it is at the equivalent ‘head organizer” site in the *Hydra* polyp [81]–[83]. Co-expression of orally expressed putative pathway inhibitors such as *Clytia* NotumO is consistent with a role in limiting the extent of Wnt activity, equivalent to its action in *Drosophila* imaginal discs [76] or during planarian head regeneration [84]. Most of the putative Wnt antagonists we identified, however, were expressed aborally in the gastrula and in aboral pole subdomains in the planula (demonstrated by *in situ* hybridization for Dkk1/2/4, Dan1, sFRP-A and NotumA, implied by DGE responses for sFRP-B and MESD), suggesting that Wnt signaling is inhibited actively at the aboral pole region in the larva. Future functional studies will be required to examine the functions of each Wnt regulator during *Clytia* development, and to unravel the interactions between them.

### New candidate developmental regulators of potentially wide interest

Our study uncovered many potential developmental regulators amongst gene families with orthologs and/or shared domains identifiable from the mass of available genomic and transcriptomic data across bilaterian species, but for which nothing is known about function or expression. These include zinc finger and helix-loop-helix domain transcription factors as well as putative novel signaling pathway components. The prominence of cell surface protein modifiers with known impact on one or several signaling pathways in our screen raises the possibility that some of the other uncharacterized conserved or cell surface proteins may function similarly. In this context it would be interesting, for example, to test the function of the ZpdA and Aat genes, which code for a likely cell surface glycoprotein and a membrane transport protein respectively. Uncovering developmental roles for such proteins in *Clytia* would open the way to explore the involvement of potential novel regulators of key embryonic and cellular processes in bilaterians, and the associated evolutionary and medical implications. WegA1 offers an interesting illustration of this possibility. WegA1-MO injection results in a spectacular developmental defect involving premature cell ingression (a process of epithelial-mesenchymal transition) at gastrulation, and a massive shift in the balance of ectoderm to endoderm formation. This finding implies that this previously unknown protein functions during normal development under the control of Wnt/β-catenin and PCP signaling to inhibit cell ingression in aboral territories. As well as the 135-amino acid, C-terminal DUF3504 domain the WegA1 sequence contains a putative nuclear localization signal. Whether it has true orthologs in bilaterians remains to be established.

### Cnidarian restricted genes

Amongst the potential developmental regulators identified in our study, 29% were defined as cnidarian-restricted on the basis that they had no identifiable orthologs in any other metazoans. Previous surveys of available cnidarian genomic and transcriptomic data revealed about 25% in *Clytia* and 15% in the ‘polyp only’ cnidarian models *Nematostella* and *Hydra* [4], [14], [18], [23]. A few of these match genes previously known only outside Metazoa, and so represent ancient genes lost in bilaterian branches or gained by lateral gene transfer, while the others probably represent cnidarian innovations. Although more in depth studies of each gene are required, the characteristic phenotypes observed in our morpholino experiments support the stereotypical expression pattern data in suggesting roles in regulating developmental processes for these cnidarian-restricted genes: larval oral pole organisation for WegO1, endoderm formation for WegIE2 and epithelial organization for WegD1 respectively.

More than half of the cnidarian-restricted transcripts identified in our study contained secretion signal sequences. These are prime candidates for roles in cell-cell signaling, either as ligands or as modulators of ligand/cell surface/receptor interactions during axis establishment and gastrulation. Candidate receptors for such signaling molecules include the many unclassified 7tm receptors identified particularly amongst IE profile/DGE class 2 transcripts. With notable exceptions such as Frizzled and Patched, members of the 7tm superfamily, including the G–protein coupled receptors (GPCRs), have not been strongly implicated in developmental regulation in bilaterians. This family has expanded independently in cnidarians [14], so its exploitation for developmental signaling might represent a cnidarian specialty, a fascinating possibility to explore in future studies.

Intriguingly, almost all (35/37) of the cnidarian-restricted genes we identified belonged to the three DGE classes associated with regional expression and thus embryo patterning at the gastrula stage (Figure 10). Conversely, the DGE Class 4 transcript set contained a higher proportion of broadly conserved “ancient” genes. Recent studies have demonstrated that the extensive variation in modes of early embryogenesis between species correlates with expression of evolutionarily “newer” genes, while subsequent ‘phylotypic stages’ (corresponding to neurula and somatogenic stages in vertebrates and the germ-band segmentation stage in insects) are strongly conserved at the phylum level and tend to express more ancient genes [85], [86]. With the caveat that our analysis concerns only a small fraction of the transcriptome and provides only limited coverage of developmental stages, the observation that most (28/35) of the DGE class 1–3 (putative patterning) genes lacked counterparts in *Nematostella* or *Hydra* may reflect the widely divergent modes of early embryo patterning and gastrulation amongst cnidarian species [87]. In contrast several of the DGE class 4 genes, mostly “ancient”, appeared to be associated with epithelia development and in particular with formation of the basal lamina, a structure considered to be a major innovation in the animal lineage [88], [89] and highly conserved in all Eumetazoa. A temporal shift in expression from “new” to “old” genes between gastrula and larva in cnidarian species is consistent with the idea that the epitheliarized, planula stage-ciliated torpedo larva represents the phylotypic stage [90].

To conclude, from a methodological standpoint, our study demonstrates the power of rigorous unbiased transcriptomic approaches to obtain a fresh view of gene conservation and innovation in the evolution of animal diversity. It also illustrates how transcriptome comparisons can allow prediction of expression characteristics without doing large-scale *in situ* hybridization screens; The differential transcriptional responses in Fz1-MO and Stbm-MO embryos will be very useful for picking candidate genes for future studies targeted to particular developmental processes. From a theoretical standpoint, our findings provide strong support for the notion that many evolutionary-conserved genes are deployed across eumetazoans to regulate development, but also good evidence that developmental regulation in cnidarians may involve a significant number of taxon-restricted genes. Functional studies of the genes identified here in *Clytia* should provide a fruitful entry for exploring both these possibilities.

## Materials and Methods

### Embryo manipulation, culture and harvesting

Eggs obtained by light-induced spawning of laboratory-raised medusae were microinjected with morpholino oligonucleotides prior to fertilization as described [39]. Previously unpublished morpholino sequences are provided in File S7. Use of genetically identical female medusae derived from a single individual laboratory polyp colony Z^4^B and males from a closely related colony [32] restricted the problems of sequence polymorphism. After culture at 18°C to the four cell stage, any unfertilized or abnormally-dividing embryos were removed. Early gastrula stage embryos, used for RNA extraction or fixed for *in situ* hybridization or confocal microscopy, were obtained after culture at 16°C overnight (17 hours). Planulae were fixed for *in situ* hybridization after 24 or 48 hours of culture at 18°C. Particular care was taken to use identical timing and temperature regimes for all experiments.

### DGE analysis

For each experimental condition, total RNA was extracted from batches of 900–1400 early gastrula stage embryos using RNAqueous kit (Life Technologies/Ambion, CA). RNA integrity was confirmed by formaldehyde gel electrophoresis. Two independent biological replicates were performed for the uninjected and Wnt3-MO conditions, and single samples for the other morpholino conditions. Estimated final embryo numbers in each sample, after removal of any showing arrested cleavage or irregular development, were as follows: Uninjected: each 1900; Wnt3-MO: each 2300, Fz1-MO: 900, Fz3-MO: 1600 and Stbm: 1400. Library construction and Illumina short-read (51 bp) sequencing was performed by GATC (Konstanz, Germany).

To quantify gene expression, the number of mapped reads onto a reference transcriptome data set was taken as a measure of transcript level. The reference transcriptome, comprising 24893 distinct (non-overlapping) assembled sequences, was built by combining, using CAP3 software, previous EST data [15], [32] and Illumina sequences from one of the untreated early gastrula samples generated in this study. Redundant sequence entries were eliminated by USEARCH (ver. 5.2.32_i86linux32). The longest predicted ORF from each sequence was used as the reference for read mapping. To reduce polymorphism, adaptator sequences and probable 5′ UTR sequences upstream of the first ATG in each cDNA contig were removed,

For each experimental condition approximately 80 million of 51pb Illumina reads were mapped on the reference transcriptome using the Bowtie command, with tolerance of two mismatches. Reads that matched to more than one reference sequence were not taken into account. Around 35% of the reads obtained for each condition could be mapped using this method. Statistical analysis was performed using the DEGseq R package [50] to determine for each transcript whether the observed ratio of transcript levels (M) between two samples is significant given the global average expression (A). The Random Sampling Model employed assumes a normal distribution for log_2_(C), where C is the number of counts, as confirmed for our data by a Q-Q plot (Figure 1B). M = log_2_(C sample1)-log_2_(C sample2) estimates the difference of expression between the conditions; A = (log_2_(C sample1)+log_2_(C sample2))/2 measures the average expression in the two conditions. A p-value was generated for each gene to determine whether the expression difference between samples was significant. A z-score was generated for each transcript, as a measure of the deviation from the random model (z-score = (Mobserved – Mexpected according to random sampling model)/Var(M expected according to random model). The MATR method used an estimation of the variation between duplicate embryo samples (calculated using the CTR method) to generate a second MA plot and to adjusts the z-score accordingly.

### Gene sequence analysis

A R-script was devised to analyze automatically the six possible reading frames of each unique assembled transcript sequence and to predict the best ORF (“find. ORF” script downloadable at http://octopus.obs-vlfr.fr/R_scripts). Sequence comparisons were performed with both BLASTx with the whole sequence and BLASTp with predicted translated ORF against the “non-redundant’” (nr) NCBI database. Domain analyses (Files S1 and S3) were performed using Interproscan, SignalP for the detection of secreted peptide signals and TMHMM for the prediction of transmembrane domains. Gene identities (column 3 of File S1) were based on BLAST and domain analyses. Gene accession numbers are provided in File S1 and File S3.

### Orthology analysis

To determine orthology of the transcript sequences studied in detail (Table 1) we searched for homologs by reciprocal BLASTp. When reciprocal blast and domain analysis (see above) gave unambiguous identities (non-multigene families), gene names were attributed directly (Sulf, Aat, Asparaginase, Amt, UCP). For certain multigenic developmental regulator families, we added our candidate sequence and the retrieved cnidarian sequences to alignments from previously published studies kindly provided by authors (see File S2 and acknowledgements). Where no existing appropriate alignments were available, sequences from a range of eumetazoan genomes (*Drosophila melanogaster, Lottia giganta, Strongylocentrotus purpuratus, Xenopus laevis or Homo sapiens, H. magnipapillata and N. vectensis*) were aligned using MUSCLE, the best fitting model of evolution was determined using ProtTest2.4, and phylogenetic analysis performed using PhyML3.0. The trees are available in File S2.

In cases where clear *Hydra magnipapillata* orthologs were identified, further analysis was performed using the *Hydra vulgaris* transcript dataset (HAEP) available at http://compagen.zoologie.uni-kiel.de/blast.html) [91]. The number of matching reads recorded in each separated cell population (endoderm, ectoderm and *nanos*-positive cells) was normalized with respect to total read number (File S5).

For cnidarian-specific sequences, WegO1, WegO2, WegIE2, WegA2, WegD2, Zpd, had no recognisable homologs in *Hydra* or in *Nematostella* genomes. For WegIE1, WegD1 we identified single orthologs in Hydra: (listed in File S5).

### Gene cloning and probe synthesis

*In situ* hybridization probes were synthesized from cDNA clones corresponding to our EST collection when available. For the remaining sequences, cDNAs were cloned by PCR using the TOPO-TA cloning kit (Invitrogen). All sequences were verified before probe synthesis. DIG-labeled antisense RNA probes for *in situ* hybridization were synthesized using Promega T3/T7/Sp6 RNA polymerases, purified using ProbeQuant G-50 Micro Columns (GE Healthcare) and taken up in 100 µl of 50% formamide.

### *In situ* hybridization

Gastrulae, 24hpf and 48hpf planula larvae were fixed in 3.7% formaldehyde/0.2% glutaraldehyde in PBS for 2 hours on ice, washed five times in PBST (PBS containing 0.1% Tween 20) for 10 minutes, dehydrated in PBST/50% methanol and stored in methanol at −20°C. *In situ* hybridization was performed using the InsituPro robot (Intavis). After rehydratation in PBST/50% methanol and three 5 minute washes in PBST, samples were transferred to the plate. The robot program was as follows: two 20 min washes in PBST; 20 min in PBST/50% hybridization buffer (5X SSC, 50% deionized formamide, 1% dextran sulfate, 1% SDS, 50 µg/ml tRNA, 50 µg/ml heparin); 20 min in hybridization buffer; 2 hours pre-hybridization in hybridization buffer at 62°C; 40 to 63 hours hybridization at 62°C with the denatured DIG-labelled RNA probe; four 30 min washes in 5X SSC, 0.1% Tween 20 and 50% formamide at 62°C; four 30 min washes in 2X SSC, 0.1% Tween 20 and 50% formamide at 62°C; two 20 min washes in 2X SSC, 0.1% Tween 20 at 62°C; two 20 min equilibration steps in MABT (100 mM maleic acid pH 7.5, 150 mM NaCl, 0.1% Triton X-100); 1 hour blocking in MABT/1% blocking reagent (Roche); 3 hours incubation with an alkaline phosphatase labeled anti-DIG antibody diluted 1/2000 in the blocking solution; seven 20 min washes in MABT; three 20 min washes in TMNT (100 mM Tris-HCl pH 9.4, 50 mM MgCl_2_, 100 mM NaCl and 0.1% Tween 20). The color reaction was performed manually in TMNT containing 0.08 mg/ml NBT and 0.1 mg/ml BCIP (Promega). Color development time varied from 1 hour to 1 day. Samples were then washed twice in water, three times in PBS, post-fixed in PBS/3.7% formaldehyde and washed three times with PBST before mounting in 40% glycerol.

### Gene function analysis and microscopy

For the selected candidate genes we addressed phenotype specificity by designing and testing several morpholinos targeting different parts of the sequence, discarding any that proved toxic to cell division during pre-gastrula development. We could only identify 1 non-toxic morpholino targeting FoxQ2c and WegA1, and none for FoxQ2a. For Bra2 one morpholino targeted the predicted AUG translation initiation codon and the other an exon-intron junction (all details in File S7). For each morpholino we first injected a range of concentrations into eggs prior to fertilization, and then assessed planula morphology for the lowest non-toxic dose at 24 h and 48 h. The cellular basis of the observed phenotypes was then further assessed by confocal microscopy. Images of *in situ* hybridization profiles and DIC images of live embryos were acquired on an Olympus BX51 microscope. For confocal imaging of cell boundaries using fluorescent phalloidins and nuclei using Hoechst 33358 or TOPRO-3 dyes, embryos were fixed, processed and imaged on a Leica SP5 microscope as described previously [39]. Microtubules were stained by immunofluorescence using anti-α tubulin rat monoclonal antibody YL1/2 (Sigma) followed by rhodamine-conjugated anti-rat Ig antibodies (Jackson Immunoresearch).

### Quantitative RT-PCR

Total RNA from 60 Wnt3-MO injected and 60 non-injected early gastrulae was extracted using RNAqueous-Micro kit according to the manufacturer's instructions (Ambion, Warrington, UK). Genomic DNA was removed by a DNAse I treatment (Ambion) and this step was controlled for each RNA extract. First-strand cDNA was synthesized using 500 ng of total RNA, Random Hexamer Primers and Transcriptor Reverse Transcriptase (Roche Applied Science, Indianapolis, USA). Quantitative PCRs were run in quadruplicate and EF-1alpha used as the reference control gene. Each PCR contained 5 µl cDNA 1/400, 10 µl SYBR Green I Master Mix (Roche Applied Science), and 200 nM of each gene-specific primer, in a 20 µl final volume. PCR reactions were run in 96-well plates, in a LightCycler 480 (Roche Applied Science). Sequences of forward and reverse primers designed for each gene: EF-1alpha-F 5′ TGCTGTTGTCCCAATCTCTG 3′; EF-1alpha-R 5′ AAGACGGAGTGGTTTGGATG 3′; Bra-F 5′ GCAACACCCACAACAACAAC 3′; Bra-R 5′ TACGGGAAACATACGCCTTC 3′; NotumO-F 5′ GGGACATCTAAAACCCATGC 3′; NotumO-R 5′ CATGGATCTCGCATTGTGAC 3′; ZnfO-F 5′ TGCTGCTAACAACGACCAAC 3′; ZnfO-R 5′ TGGTGGAAGTGGAGATTGTG 3′; Mos3-F 5′ ATCTTACGTCCCGAACAACG 3′; mos3-R 5′ ATCCACCAATGGCAGCTTAC 3′; Znf845-F 5′ AGACCGACAGCATTTCATCC 3′; Znf845-R 5′ TGGCATTCCTTGCATACCTC 3′; Dkk1/2/4-F 5′ GGGCTTGTTCGTACTTTTCC 3′; Dkk1/2/4-R 5′ ATTCCATCCCACGACAACAC 3′; ZnfA-F 5′ CAACAACTTTCACCGAGCTG 3′; ZnfA-R 5′ TGTCTCTTGTGTTGCCAAGC 3′; Dan1-F 5′ CATGCCCGTTCATGAGAAAG 3′; Dan1-R 5′TTTTGGCTGTTCCCACTGTC 3′; NotumA-F 5′ TGCTGAAGGGTCGTACATTG 3′; NotumA-R 5′ CGTGTGTCCATTTTCAGTGC 3′; HD02-F 5′ TT AACAGCCCACCGAAACTC 3′; HD02-R 5′ CGTCGTGTTTTTCAGTGACG 3′.

For each gene studied an expression level N was calculated as 2^−Ct^, where Ct (Cycle threshold) represents the number of cycles required for the fluorescent signal to cross the threshold.

## Supporting Information

File S1 Primary list of transcripts differentially expressed in Wnt3-MO compared to non-injected embryos identified by DGE (z-score cutoff of +/− 5.0), with putative identities, domain analysis, z-scores calculated for three MO–injected embryo conditions, accession numbers and DGE classes assigned in relation to Wnt3-MO and Fz1-MO responses (see text).
(XLS)

File S2 Phylogenetic analyses (using PhyML) to determine the orthology of the *Clytia* sequences studied in detail in this study : Antp family (HD02, Six4/5 and Gsc) ; T-box family (Tbx, Bra1, Bra2); Pax Family (PaxA), Notum family (NotumA, NotumO); Botch Family (Botch1 and Botch2); Dkk; DMRT-E; Forkhead family (FoxA, FoxC, FoxQ2a, FoxQ2b, FoxQ2c,); Frizzled/sFRP family (sFRP-A, sFRP-B, Fz1, Fz2, Fz3, Fz4); Dan family (Dan1); Hlh family (HlhIE1; HlhIE2); Mos family (Mos1, Mos2, Mos3); Erg; Myb; Znf subfamilies for ZnfO, ZnfA and Znf845. For bZip, Asp, Amt, Sulf, phylogenetic analysis was not performed since only one *Clytia* homolog was found. *Hydra* orthologs are provided in File S5. Note that only the *Clytia* genes featured in this study were included, not other related *Clytia* sequences. See Materials and Methods for details of the datasets used.
(DOC)

File S3 Secondary list of differentially expressed sequences in Wnt3-MO compared to non-injected embryos identified by DGE with z-scores outside the +/− 5.0 cutoff but greater than +3.3/less than −3.3.
(XLS)

File S4 Expression patterns of additional selected transcripts. *In situ* hybridization profiles for FoxQ2c (A); NotumO (B); WegO3 (C); Goosecoïd (D); sFRP-A (E) and Tbx (F). FoxQ2c shows an IE-type pattern, NotumO, WegO3 and Goosecoïd show O-type patterns, sFRP-A and Tbx show A-type patterns. The correlation with DGE responses holds in all cases (see Table 1). Scale bars and panel organization as in Figures 2 and 3.
(TIF)

File S5 *Hydra* orthologs of the *Clytia* gene analyses from genomic and transcriptomic data, along with information where available on differential expression between *Hydra* tissues [25]. Orthology was confirmed by phylogenetic (PhyML) analysis of sets of closely related sequences from cnidarians.
(XLS)

File S6 *In situ* hybridization analysis of selected D-type pattern transcripts in untreated (left panels) and Wnt3-MO injected (right panels) early gastrulae. A: ZpdA transcripts; B: Botch2 transcripts, C: Asp transcripts. Cells with high expression on the blastocoelar face of the ectoderm were discernible (narrows). All control embryos are oriented with the oral pole uppermost. Gene identity is shown in the bottom right of each pair of panels. Scale bar 50 µm.
(EPS)

File S7 Details of all previously unpublished morpholinos used in this study, including injection doses and sequences.
(XLS)

File S8 Confocal images of control and morpholino embryos fixed after about 48 hpf and phalloidin stained for the visualisation of cell contours (A-H, L, M, P-R) or anti-tubulin immunofluorescence (red) and nuclear staining with Hoechst or TOPRO-3 dyes (blue) (I-K, N, O, S). Endogenously expressed GFP-1 [92] provided a marker of the planula ectoderm (green in P, T, U). Oral poles are at the top in all images. Uninjected planula larvae (A) showed well-defined, epithelialized ectoderm and endodermal layers. WegO1 (B), Bra1 (C) and Bra2 (D) showed deficits of endoderm with regions of empty blastocoel still found (asterisks) as well as residual ectodermal cells accumulating towards the aboral pole (white arrowheads). This defect was much less severe in WegO1-MO embryos. FoxQ2c-MO (E) and WegIE2-MO (F) embryos showed severely disrupted endodermal layers (black arrows). WegA1-MO injection generated aggregates of endodermal-like cells of variable sizes covered in some (G, I) but not all (H) cases by a thin layer of ectodermal cells. HD02-MO (L-P) and WegD1-MO (Q-T) embryos had severely disrupted morphology characterized by a disruption of the basal lamina between the endoderm and GFP-expressing ectoderm layers (white arrows in P and T; compare with the uninjected planula in U). The WegD1-MO embryos were filled with disorganized cell sheets and lacked the central stripe of cell destruction characteristic of normal endodermal cavity formation. Anti-tubulin staining revealed disorganized bundles of neurite-like processes in the ectoderm-endoderm interface in HD02-MO embryos contrasting with the more orderly organization in uninjected embryos (pink arrows in confocal images in O and K respectively, acquired at this level) and the lack of this layer in WegD1-MO embryos (S). Scale bar 50 µm.
(TIF)
