# Correction: Differential Responses to Wnt and PCP Disruption Predict Expression and Developmental Function of Conserved and Novel Genes in a Cnidarian

**DOI:** 10.1371/journal.pgen.1004781

**Published:** 2014-10-17

**Authors:** The PLOS Genetics Staff

## Notice of Republication

This article was republished on September 23, 2014, to correct an error in the way Figure 3 was displayed online. The pdf was and is still correct.

## References

[1] Lapébie P, Ruggiero A, Barreau C, Chevalier S, Chang P, et al (2014) Differential Responses to Wnt and PCP Disruption Predict Expression and Developmental Function of Conserved and Novel Genes in a Cnidarian. PLoS Genet 10(9): e1004590 doi:10.1371/journal.pgen.1004590 25233086 10.1371/journal.pgen.1004590 PMC4169000

