# Supplementary material for: Differential Responses to Wnt and PCP Disruption Predict Expression and Developmental Function of Conserved and Novel Genes in a Cnidarian
Source: PLoS Genet. 2014 Sep 18;10(9):e1004590. doi: 10.1371/journal.pgen.1004590 (PMC4169000; doi:10.1371/journal.pgen.1004590)
Supplement: File S2 — Phylogenetic analyses (using PhyML) to determine the orthology of the Clytia sequences studied in detail in this study : Antp family (HD02, Six4/5 and Gsc) ; T-box family (Tbx, Bra1, Bra2); Pax Family (PaxA), Notum family (NotumA, NotumO); Botch Family (Botch1 and Botch2); Dkk; DMRT-E; Forkhead family (FoxA, FoxC, FoxQ2a, FoxQ2b, FoxQ2c,); Frizzled/sFRP family (sFRP-A, sFRP-B, Fz1, Fz2, Fz3, Fz4); Dan family (Dan1); Hlh family (HlhIE1; HlhIE2); Mos family (Mos1, Mos2, Mos3); Erg; Myb; Znf subfamilies for ZnfO, ZnfA and Znf845. For bZip, Asp, Amt, Sulf, phylogenetic analysis was not performed since only one Clytia homolog was found. Hydra orthologs are provided in File S5. Note that only the Clytia genes featured in this study were included, not other related Clytia sequences. See Materials and Methods for details of the datasets used. (DOC) [file pgen.1004590.s002.doc]

# Antp family: HD02, Six4/5 and Gsc.

The alignment of homeodomain has been adapted from [1]

Following cnidarian sequences have been added:

(HmaAntp01 :ABB86456.1) (HmaAntp02 :XP_002168431.2) (HmaAntp04 :XP_002162385.2) (HmaAntp05 :AFK74886.1) (HmaAntp06 :XP_002153848.2) (HmaAntp07 :AAB58291.1) (HmaAntp08 :) (HmaAntp09 :XP_004206594.1) (HmaAntp10 :XP_002163770.1) (HmaAntp11 :XP_002156512.1) (HmaAntp12 :XP_002165751.1) (HmaAntp13 :XP_004206396.1) (HmaAntp14 :XP_002163407.1) (HmaAntp15 :XP_002163407.1) (HmaAntp16 :XP_002156835.1) (HmaAntp17 :XP_002163595.1) (HmaAntp18 :ADB66163.1) (HmaAntp19 :XP_002162903.1) (HmaAntp20 :XP_002161581.1) (HmaAntp21 :XP_002160735.2) (HmaAntp22 :XP_002157088.1) (HmaAntp23 :AAD32576.1) (HmaAntp24 :XP_002159502.1) (HmaAntp25 :AAD30998.1) (HmaAntp26 :CAA75669.1) (HmaAntp27 :XP_002163599.1) (HmaAntp28 :XP_002156720.2) (HmaAntp29 :XP_002164410.2) (HmaAntp30 :XP_002164986.2) (HmaAntp32 :XP_002160848.1) (HmaGsc :AAF14575.1) (Nv_002 :ABG67881.1) (Nv_003 :ABB83731.1) (Nv_004 :ABB29576.1) (Nv_005 :ABB83732.1) (Nv_006 :XP_001634250.1) (Nv_007 :XP_001633591.1) (Nv_009 :ABB86425.1) (Nv_010 :ABB86464.1) (Nv_011 :ABB83732.1) (Nv_012 :XP_001634252.1) (Nv_013 :XP_001640773.1) (Nv_014 :ABB83735.1) (Nv_015 :ABB86467.1) (Nv_017 :XP_001631266.1) (Nv_018 :XP_001634533.1) (Nv_019 :ABB86470.1) (Nv_020 :ABB86471.1) (Nv_021 :ABB86472.1) (Nv_022 :ABB83736.1) (Nv_023 :XP_001630315.1) (Nv_024 :ABB29577.1) (Nv_025 :ABB86426.1) (Nv_026 :XP_001619733.1) (Nv_027 :ABB86427.1) (Nv_028 :ABB29578.1) (Nv_029 :ABB86475.1) (Nv_030 :ABB86428.1) (Nv_031 :ABB86429.1) (Nv_032 :ABB86430.1) (Nv_033 :XP_001630954.1) (Nv_034 :AFJ11249.1) (Nv_035 :ABB86478.1) (Nv_036 :ABC16640.1) (Nv_037 :ABB72462.1) (Nv_038 :ABB83732.1) (Nv_040 :ABB83738.1) (Nv_041 :ABB86431.1) (Nv_042 :ABB86479.1) (Nv_043 :ABB86432.1) (Nv_044 :ABB86480.1) (Nv_045 :ABB83739.1) (Nv_046 :ABG67794.1) (Nv_047 :ABB83740.1) (Nv_048 :ABB86481.1) (Nv_049 :ABB86482.1) (Nv_050 :ABB86483.1) (Nv_051 :ABG67778.1) (Nv_052 :ABB86435.1) (Nv_054 :XP_001635500.1) (Nv_055 :BAH58093.1) (Nv_056 :XP_001635465.1) (Nv_057 :ABB86484.1) (Nv_058 :ABB83741.1) (Nv_059 :ABB72463.1) (Nv_060 :ABB86485.1) (Nv_061 :ABB86486.1) (Nv_063 :XP_001630728.1) (Nv_064 :ABB83731.1) (Nv_065 :ABC16641.1) (Nv_066 :ABB86439.1) (Nv_067 :ABB86487.1) (Nv_069 :XP_001634306.1) (Nv_070 :XP_001622982.1) (Nv_071 :ABB86489.1) (Nv_072 :ABB86441.1) (Nv_073 :ABB86490.1) (Nv_074 :ABB83744.1) (Nv_075 :ABB86491.1) (Nv_076 :XP_001630315.1) (Nv_077 :XP_001628939.1) (Nv_078 :ABB83745.1) (Nv_079 :ABB72465.1) (Nv_080 :XP_001641364.1) (Nv_081 :ABB86494.1) (Nv_082 :ABB86495.1) (Nv_083 :ABB86443.1) (Nv_084 :ABB86444.1) (Nv_085 :XP_001631223.1) (Nv_086 :ABB83746.1) (Nv_088 :ABB83747.1) (Nv_090 :XP_001641711.1) (Nv_091 :ABB83748.1) (Nv_092 :ABB86497.1) (Nv_093 :ABB86445.1) (Nv_094 :ABB86446.1) (Nv_095 :ABB86447.1) (Nv_096 :ABB86498.1) (Nv_097 :ABB86499.1) (Nv_098 :ABB86500.1) (Nv_099 :ACT36586.1) (Nv_100 :XP_001623896.1) (Nv_101 :ABB29579.1) (Nv_102 :ABB86448.1) (Nv_103 :ABB86449.1) (Nv_104 :ABB86450.1) (Nv_105 :XP_001641710.1) (Nv_106 :ABB86451.1) (Nv_107 :XP_001628502.1) (Nv_108 :XP_001634045.1) (Nv_109 :ABB86502.1) (Nv_110 :AAP88428.2) (Nv_112 :XP_001622987.1) (Nv_114 :XP_001647538.1) (Nv_115 :ABB86453.1) (Nv_116 :XP_001640830.1) (Nv_117 :ABB86454.1) (Nv_118 :XP_001631425.1) (Nv_119 :XP_001625948.1) (Nv_120 :XP_001630989.1) (Nv_121 :XP_001634225.1) (Nv_122 :XP_001634765.1) (Nv_123 :ABB83750.1) (Nv_124 :ABB86456.1) (Nv_125 :ABB86508.1) (Nv_126 :XP_001634259.1) (Nv_127 :ABB86509.1) (Nv_128 :ABB86458.1) (Nv_129 :ABB83751.1) (Nv_130 :AAK28380.1) (Nv_142 :ABG67792.1) (Nv_143 :ABC16638.1) (Nv_144 :ABC16639.1) (Nv_145 :ABB86510.1) (Nv_146 :ABB83752.1) (Nv_147 :ABB86461.1) (Nv_148 :ABB83753.1) (Nv_149 :XP_001633241.1) (Nv_151 :ABC16642.1) (Nv_170 :ABB86496.1) (Nv_171 :XP_001634250.1)


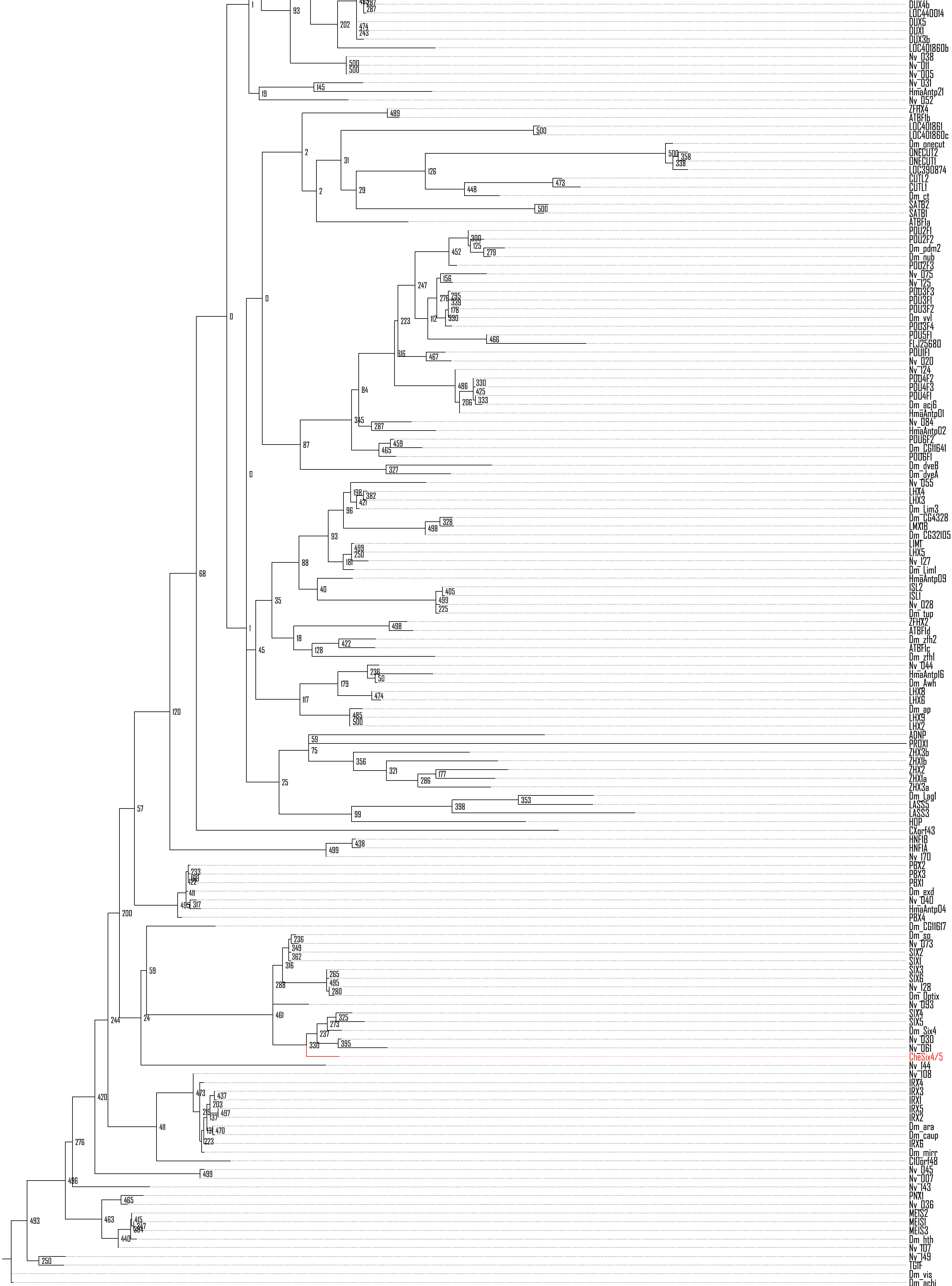


# Tbx family:

Following sequences have been identified from genomes of Capitella teleta (Cap), Ciona intestinalis (Ci), Nematostella vectensis (Nv), Mus musculus (Mm), Danio rerio (Dr), Lottia giganta (Lg), Drosophila melanogaster (Dm), Hydra magnipapillata (Hm), Branchiostoma floridae (Amphi), Xenopus laevis (Xl), Helobdella robusta (Hr).

(AmphiEomes :XP_002589092.1) (AmphiTbx1 :AAG34887.2) (AmphiTbx2 :AAG34888.1) (AmphiTbx6 :AAG34890.1) (BfTbx1518 :AAG34891.1) (Cap1241000 :ELU15946.1) (Cap190074 :ELU10823.1) (Cap1960038 :ELU10394.1) (Cap2380027 :ELU07917.1) (Cap2960003 :ELU04858.1) (Cap7420011 :ELT91126.1) (Cape_gw1_7 :ELT90467.1) (CiBra :NP_001027659.1) (CiTbx1 :NP_001027622.1) (CiTbx1518 :NP_001027588.1) (CiTbx20 :NP_001027590.1) (CiTbx23 :NP_001027620.1) (CiTbx6a :XP_002126887.2) (CiTbx6b :NP_001027752.1) (CL1Contig5 :CAD21521.1) (CL4466Cont :ELR50901.1) (CL4556Cont :CAE45765.1) (Dmbi :XP_002037013.1) (Dmbyn :NP_001261730.1) (DmCG6634P :XP_002037951.1) (DmDoc1 :NP_001261612.1) (DmDoc3 :XP_002084212.1) (DmH15 :CAA67304.1) (Dmorg1 :CAA76529.1) (DrEomesod :AAG48250.2) (Drnotail :NP_571237.1) (Drsimilar :XP_003198807.1) (DrT-box15 :XP_008064094.1) (DrT-box18 :AAI54827.1) (DrT-box24 :Q8JIS6.2) (Drtbx1 :ELR50901.1) (Drtbx16 :NP_571133.1) (Drtbx2 :XP_005807793.1) (Drtbx20 :NP_571581.1) (Drtbx4 :NP_570989.1) (Drtbx5 :AAF22296.1) (Drtbx6 :XP_005165323.1) (HmBra1 :AAD26626.1) (HmBra2 :XP_002154016.2) (HrTbxA :ESO11613.1) (HrTbxB :ESO06717.1) (HrTbxC :ESN92283.1) (HrTbxD :ESO03108.1) (HrTbxG :ESO04968.1) (HrTbxH :ESO05344.1) (HrTbxI :ESO01901.1) (HrTbxJ :ESO08589.1) (HrTbxK :ESN99217.1) (HrTbxL :ESN98407.1) (HrTbxN :ESN92282.1) (HrTbxO :ESN93190.1) (HrTbxP :ESO08164.1) (LgTbr :ESO86062.1) (LgTbxA :ESO97556.1) (LgTbxB :ESO95446.1) (LgTbxC :ESO95443.1) (LgTbxD :ESO95444.1) (LgTbxE :ESO86498.1) (LgTbxF :ESO86062.1) (LgTbxG :ESO87302.1) (LgTbxH :ESP04123.1) (LgTbxI :ESO86499.1) (MmEomes :EFB16237.1) (MmT :ABM91941.1) (MmTbr1 :XP_007421591.1) (MmTbx1 :ELR50901.1) (MmTbx10 :NP_035663.1) (MmTbx15 :XP_008064094.1) (MmTbx18 :XP_006715665.1) (MmTbx19 :EGW08782.1) (MmTbx2 :EDL15776.1) (MmTbx20 :CAB51916.1) (MmTbx21 :EHH24744.1) (MmTbx22 :XP_006528098.1) (MmTbx3 :1H6F) (MmTbx4 :XP_007438843.1) (MmTbx5 :2X6U) (MmTbx6 :XP_006507617.1) (NvBra :AAO27886.2) (Nvtbx1 :AAQ23383.1) (NvTbx15b :XP_001638727.1) (NvTbx20 :XP_001629268.1) (NvTbx23 :XP_001633951.1) (CheBra2 :XP_002154016.2) (CheTbx :XP_001633952.1) (CheBra1 :ABJ16449.1) (XBRA :1XBR) (Xleomes :AAI25987.1) (Xltbx1 :ELR50901.1) (Xltbx2 :BAD92615.1) (Xltbx20 :NP_001079332.1) (Xltbx3 :1H6F) (Xltbx5 :ABV54787.1) (Xltbx6 :BAC20262.1) (XlTbxA :XP_008064094.1) (XlTbxB :XP_002933971.1) (XlTbxC :XP_002933783.2) (XlVEGT :CAA68179.1)

# Pax family

The alignment of Paired from [2]

# Notum family

Following sequences have been identified in Nematostella vectensis (Nv), Strongylocentrotus purpuratus (Sp), Lottia giganta (Lg), Drosophila melanogaster (Dm), Xenopus tropicalis (Xt) and Homo sapiens (Hsa) genomes.

Has:NP_848588.3; Xt:NP_001120228.2; Xt:AAI60399.1; Xt:XP_002932265.2, Dm:NP_730096.2; Lg:ESO88928; Sp:XP_794410.2; Sp:XP_001180113.1; (Nv21651:XP_001631492.1)(Nv110685:XP_001631492.1)(Nv86403:XP_001639461.1)(Nv21658:XP_001639461.1)(Nv209762:XP_001630961.1)(Nv88708:XP_001638718.1)(Nv21603:XP_002160336.2)

# Botch family

Following sequences have been identified in Nematostella vectensis (Nv), Hydra magnipapillata (Hm), Strongylocentrotus purpuratus (Sp), Lottia giganta (Lg), Drosophila melanogaster (Dm), and Homo sapiens (Hsa) genomes.

NvFoxG1:XP_001627122.1

NvFoxQ2a:AGD98927.1

NvFoxAB:XP_001631592.1

NvFoxQ2b:XP_001626709.1

NvFoxG2:XP_001634717.1

NvFoxD3:XP_001637167.1

NvFoxA:AAS13442.1

NvFoxB:ABA03229.1

NvFoxC:ABA03230.1

NvFoxD1:XP_001634161.1

NvFoxD2:XP_001637168.1

NvFoxE:XP_001637165.1

NvFoxL2:XP_001639875.1

NvFoxNx:XP_001641147.1

NvFoxO.1:XP_001623177.1

NvFoxO.2:DAA34163.1

HmaFoxB:XP_002166230.2

HmaFoxO:AFQ20829.1

HmaFoxN:XP_002166457.1

HmaFox2:AFK74873.1

HmaFoxJ2:XP_002156863.2

HmaFoxJ1:XP_002158068.1

HmaFoxP:XP_002164110.2

HmFoxA:AAO92606.1

HmFoxK:AFK74878.1

HmFoxQ2a:XP_002165467.2

HmFOXQ2b:XP_002159677.1

HmFoxQ2c:XP_002166935.1

# Dkk family

Following sequences have been identified in Nematostella vectensis (Nv), Hydra magnipapillata (Hm), Strongylocentrotus purpuratus (Sp), Lottia giganta (Lg), Drosophila melanogaster (Dm), and Homo sapiens (Hsa) genomes.

(cinDkk3:XP_002131545.1) (CheDkka:XP_002167329.2) (CheDkkb:CAJ65509.1) (HmDkk7:NP_001267765.1) (HmDkk5:XP_002164036.2) (HmDkk1:XP_004207403.1) (NveDkkb:XP_001642082.1) (NveDkkd:XP_001624521.1) (CheDkke:ABY71253.1) (HmDkk4:NP_001267744.1) (NveDkkc:XP_001641951.1) (CheDkkd:XP_002158204.1) (HmDkk3:XP_002158204.1) (CinDkkA:NP_001072031.1) (CheDkkc:XP_001634303.1) (XlDkk3:NP_001121290.1) (HsDkk3:BAG52632.1) (SpDkk1:NP_001091929.1) (BfDkk1:ABG34307.1) (HsDkk4:BAK63662.1) (XlaDkk2:EPY81184.1) (HsDkk2:EPY81184.1) (XlaDkk1:NP_001079061.1) (HsDkk1:XP_006144441.1) (CheDkkf:XP_001634303.1) (NvDkk:ABF55256.1) (NveDkka:XP_001634303.1)

# DMRT family

The alignment of DM domain from [3] has been used.

# Fox family

The alignment of Forhead domain from [4] have been used.

The following cnidarian sequences have been added:

NvFoxG1:XP_001627122.1

NvFoxQ2a:AGD98927.1

NvFoxAB:XP_001631592.1

NvFoxQ2b:XP_001626709.1

NvFoxG2:XP_001634717.1

NvFoxD3:XP_001637167.1

NvFoxA:AAS13442.1

NvFoxB:ABA03229.1

NvFoxC:ABA03230.1

NvFoxD1:XP_001634161.1

NvFoxD2:XP_001637168.1

NvFoxE:XP_001637165.1

NvFoxL2:XP_001639875.1

NvFoxNx:XP_001641147.1

NvFoxO.1:XP_001623177.1

NvFoxO.2:DAA34163.1

HmaFoxB:XP_002166230.2

HmaFoxO:AFQ20829.1

HmaFoxN:XP_002166457.1

HmaFox2:AFK74873.1

HmaFoxJ2:XP_002156863.2

HmaFoxJ1:XP_002158068.1

HmaFoxP:XP_002164110.2

HmFoxA:AAO92606.1

HmFoxK:AFK74878.1

HmFoxQ2a:XP_002165467.2

HmFOXQ2b:XP_002159677.1

HmFoxQ2c:XP_002166935.1

# Frizzled and sFRP family, CRD domain

The CRD domain alignment from [5] have been used .

# DAN family containing Cerberus and Gremlin groups

Following sequences have been identified in Nematostella vectensis (Nv), Hydra vulgaris (Hv), Strongylocentrotus purpuratus (Sp), Xenopus laevis (Xl) and Homo sapiens (Hsa) genomes.

HvXP002154:XP_002154217.1

CheCerberus:AAI03978.1

HsaDAN:NP_689867.1

HvDAN:XP_004210528.1

Hsaneuro:XP_005957120.1

HvGrml_like:CAJ80868.1

Hvgremlin-2:XP_002160711.1

Xlcerberus:AAH81277.1

Hsacerberu:NP_005445.1

Nvgremlin:ABF06563.1

Spgremlin:XP_003728013.1

Hsagremlin:XP_005374884.1

XlGremlin:NP_001083746.1

# Hlh family

The alignment from [6] have been used.


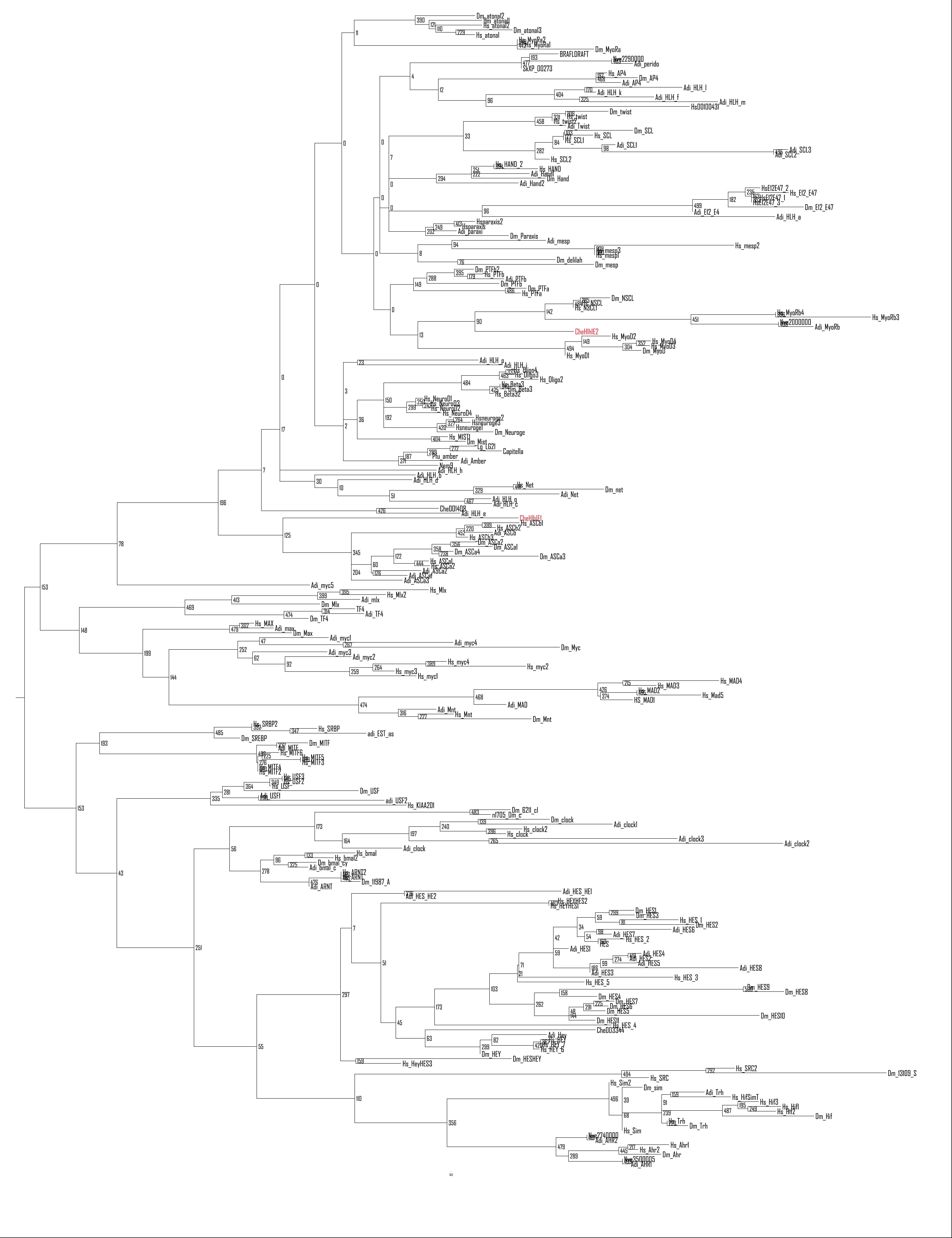


# Mos

Following sequences have been identified in Nematostella vectensis (Nv), Hydra magnipapillata (Hm), Strongylocentrotus purpuratus (Sp), Lottia giganta (Lg), Branchiostoma floridae Bf) and Xenopus tropicalis (Xt) genomes.

(CheMos1 :ACM68728.1) (XtMos :XP_004915249.1) (CtMos :ELU18820.1) (LgMos :ESO86718.1) (SpMos :XP_003729407.1) (NvMos-d :XP_001629538.1) (CheMos3 :XP_004209764.1) (HmMos :XP_004209764.1) (BfMos :XP_002611979.1) (CheMos2 :ACM68729.1) (NvMos-a :XP_001630390.1) (NvMos-c :XP_001622709.1) (NvMos-b :XP_001624116.1)

# Ets transcription

The Ets family being a large family, we chose to restrict the sequence sampling to Homo sapiens genome.

(X005268704 :ABM89342.1) (N689536.1 :ABM89342.1) (N003111.2 :XP_003920114.1) (N001074016 :XP_003920114.1) (N003112.2 :XP_003779280.1) (N001230929 :XP_003779280.1) (N001230927 :XP_003779280.1) (N001107781 :AAB65824.1) (X005252914 :ABM46710.1) (N001193545 :ABM46710.1) (X005252918 :ABM46710.1) (X005252915 :ABM46710.1) (X005252916 :ABM46710.1) (N036285.2 :ABM46710.1) (N001193544 :ABM46710.1) (N001230009 :XP_003951908.1) (N001413.1 :XP_003951908.1) (N001230010 :XP_003951908.1) (N938195.1 :XP_003951908.1) (N001978.1 :XP_007965875.1) (N001193968 :XP_003950870.1) (N001193964 :XP_003950870.1) (X005249231 :XP_003950870.1) (N001193970 :XP_003950870.1) (N001193966 :XP_003950870.1) (N001193969 :XP_003950870.1) (N001193967 :XP_003950867.1) (N001193965 :XP_003950870.1) (N057219.1 :XP_003950870.1) (X005262862 :BAH14050.1) (X005262860 :XP_003800200.1) (N973728.1 :XP_003800200.1) (X005262863 :XP_003800200.1) (N001263388 :BAH14050.1) (N001263386 :BAH14050.1) (N006865.1 :XP_003800200.1) (N001263387 :XP_003800200.1) (N001412.1 :XP_005401307.1) (N001138825 :XP_001515225.2) (N758961.1 :XP_001515225.2) (X005275903 :EHB02515.1) (N006485.2 :XP_005692640.1) (X005258700 :XP_005692640.1) (N001004341 :AAI68842.1) (N005231.1 :XP_007110376.1) (N001138784 :XP_007110376.1) (X005258711 :AAD00861.1) (X005258712 :AAI07125.1) (X005258710 :AAD00861.1) (N055024.2 :AAD00861.1) (X005258709 :AAD00861.1) (N004440.1 :4IRI) (N001129626 :4IRI) (N891548.1 :4IRI) (N001230358 :4IRI) (N001129627 :4IRI) (N001161153 :ERE74583.1) (N002008.2 :ERE74583.1) (N001257939 :ERE74583.1) (N001257941 :ERE74583.1) (N001239223 :XP_005891333.1) (N036523.1 :XP_005891333.1) (N002031.2 :XP_006751309.1) (N059991.1 :XP_003818745.1) (N005230.1 :4BQA) (N001243224 :4BQA) (N001155894 :1R36) (N005229.1 :1R36) (N001137292 :1R36) (X005271485 :1R36) (N068567.1 :1BC7) (N001964.2 :1BC7) (N005221.2 :XP_005374553.1) (N001244097 :XP_004668877.1) (N005220.2 :XP_004668877.1) (N004445.1 :XP_008056741.1) (X005247267 :XP_008056741.1) (X005247269 :XP_008056741.1) (N001248366 :NP_001248368.1) (X005257199 :NP_001248368.1) (N001977.1 :NP_001248368.1) (N001248368 :NP_001248368.1) (N001156620 :XP_007505517.1) (N001156619 :XP_007505517.1) (N004947.2 :XP_007505517.1) (N001156623 :XP_007505517.1) (N001156624 :XP_007505517.1) (N001156622 :XP_007505517.1)

# Bzip, Asp, Amt, Sulf

For these genes, no phylogenetic analysis since only one homolog has been found . (see Supporting data File S5 for ortholog in Hydra)

# Zn finger family

The Zn finger is very large. We selected the tenth best hits of blast in Hydra and Nematostella genomes in order to find an ortholog.

## ZnfO

The following sequences have been used:

Nv93710:XP_001637077

Nv85463:XP_001639816

Nv118120: XP_001629021

Nv185507:XP_001633558

Nv123933:XP_001627106

Nv105290:XP_001633145

Nv179413|e:XP_001639766

Nv82654:XP_001640902

Hm218840:XP_002167706.2

Hm213324:XP_002167706.2

Hm213324:XP_002162808.2

Hm220160:XP_002168017.2

Hm228134:XP_002157525.1

Hm229992:XP_002158983.2

Hm204384:XP_002156009.2

Hm218707:XP_002164561.2

Hm229440:XP_002156584.1

Hm230701:XP_004206018.1

## ZnfA

The following sequences have been used:

Hm44968366:XP_002164347.2

Hm22111423:XP_002155290.1

Hm44969239:XP_002167240.2

Hm44967129:XP_002167240.2

Hm44966851:XP_004206804.1

Hm44968879:XP_002170242.2

Hm44966850:XP_004206802.1

Nv107193:XP_001632514.1

Nv229565:XP_001632546.1

Nv236177:XP_001622426.1

Nv101900:XP_001634182.1

Nv27142:XP_001635004.1

Nv99612:XP_001634948.1

Hm44966857:AFK74874.1

Nv28237:XP_001635002.1

Nv82548:XP_001640812.1

Hm22112623:XP_002161066.1

Nv19278:XP_001624390.1

Nv39461:XP_001639597.1

Hm22110797:AFK74888.1

## Znf845

The following sequences have been used:

XP_0021560:XP_002156009.2

XP_0021621:XP_002162169.2

XP_0021573:XP_002157355.1

XP_0021596:XP_002159673.2

XP_0021589:XP_002158983.2

XP_0021669:XP_002166941.1

XP_0021677:XP_002167774.2

XP_0021575:XP_002157525.1

XP_0021678:XP_002162706.2

XP_0021613:XP_002161321.2

XP_0021654:XP_002165457.2

XP_0021618:XP_002161844.2

XP_0021645:XP_002164561.2

XP_0021597:XP_002159739.2

XP_0021643:XP_002164347.2

XP_0021629:XP_002162913.2

XP_0021583:XP_002158383.2

Nv1:XP_001618979.1

Nv2: XP_001621802.1

Nv5: XP_001623938.1

Nv9: XP_001630482.1

Nv10: XP_001630482.1

# References

1. Ryan J, Burton P, Mazza M, Kwong G, Mullikin J, et al. (2006) The cnidarian-bilaterian ancestor possessed at least 56 homeoboxes. Evidence from the starlet sea anemone, Nematostella vectensis. Genome Biol 7: R64–R64. doi:10.1186/gb-2006-7-7-R64.

2. Suga H, Tschopp P, Graziussi DF, Stierwald M, Schmid V, et al. (2010) Flexibly deployed Pax genes in eye development at the early evolution of animals demonstrated by studies on a hydrozoan jellyfish. Proc Natl Acad Sci 107: 14263–14268. doi:10.1073/pnas.1008389107.

3. Parlier D, Moers V, Van Campenhout C, Preillon J, Leclère L, et al. (2013) The Xenopus doublesex-related gene Dmrt5 is required for olfactory placode neurogenesis. Dev Biol 373: 39–52. doi:10.1016/j.ydbio.2012.10.003.

4. Chevalier S, Martin A, Leclère L, Amiel A, Houliston E (2006) Polarised expression of FoxB and FoxQ2 genes during development of the hydrozoan Clytia hemisphaerica. Dev Genes Evol 216: 709–720. doi:10.1007/s00427-006-0103-6.

5. Leclère L, Rentzsch F (2012) Repeated Evolution of Identical Domain Architecture in Metazoan Netrin Domain-Containing Proteins. Genome Biol Evol 4: 771–787. doi:10.1093/gbe/evs061.

6. Gyoja F, Kawashima T, Satoh N (2012) A genomewide survey of bHLH transcription factors in the coral Acropora digitifera identifies three novel orthologous families, pearl, amber, and peridot. Dev Genes Evol 222: 63–76. doi:10.1007/s00427-012-0388-6.
