# Supplementary figures and images for: Differential Responses to Wnt and PCP Disruption Predict Expression and Developmental Function of Conserved and Novel Genes in a Cnidarian
Source: PLoS Genet. 2014 Sep 18;10(9):e1004590. doi: 10.1371/journal.pgen.1004590 (PMC4169000; doi:10.1371/journal.pgen.1004590)

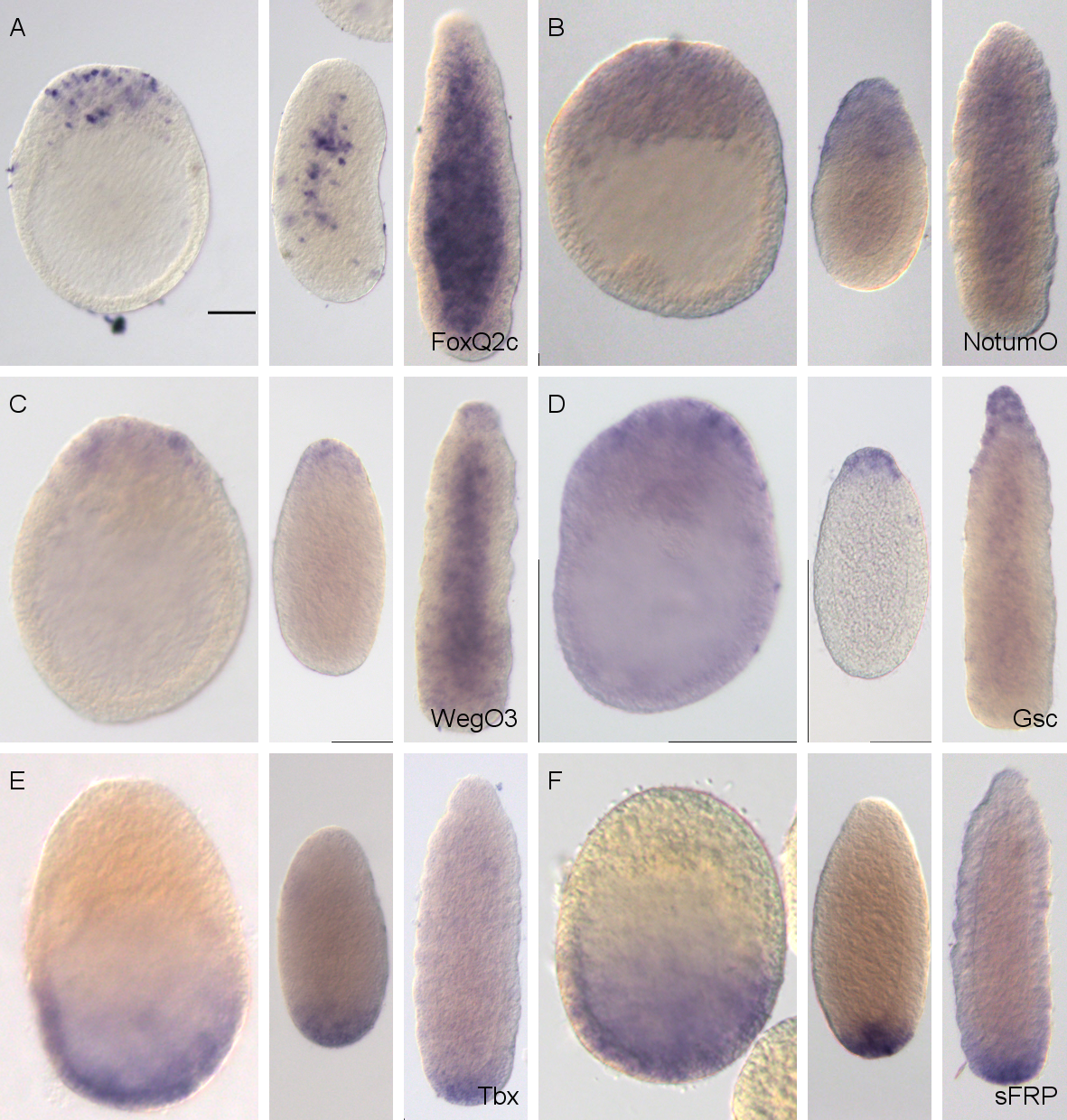

Supplement: File S4 — Expression patterns of additional selected transcripts. In situ hybridization profiles for FoxQ2c (A); NotumO (B); WegO3 (C); Goosecoïd (D); sFRP-A (E) and Tbx (F). FoxQ2c shows an IE-type pattern, NotumO, WegO3 and Goosecoïd show O-type patterns, sFRP-A and Tbx show A-type patterns. The correlation with DGE responses holds in all cases (see Table 1). Scale bars and panel organization as in Figures 2 and 3. (TIF) [file pgen.1004590.s004.tif]

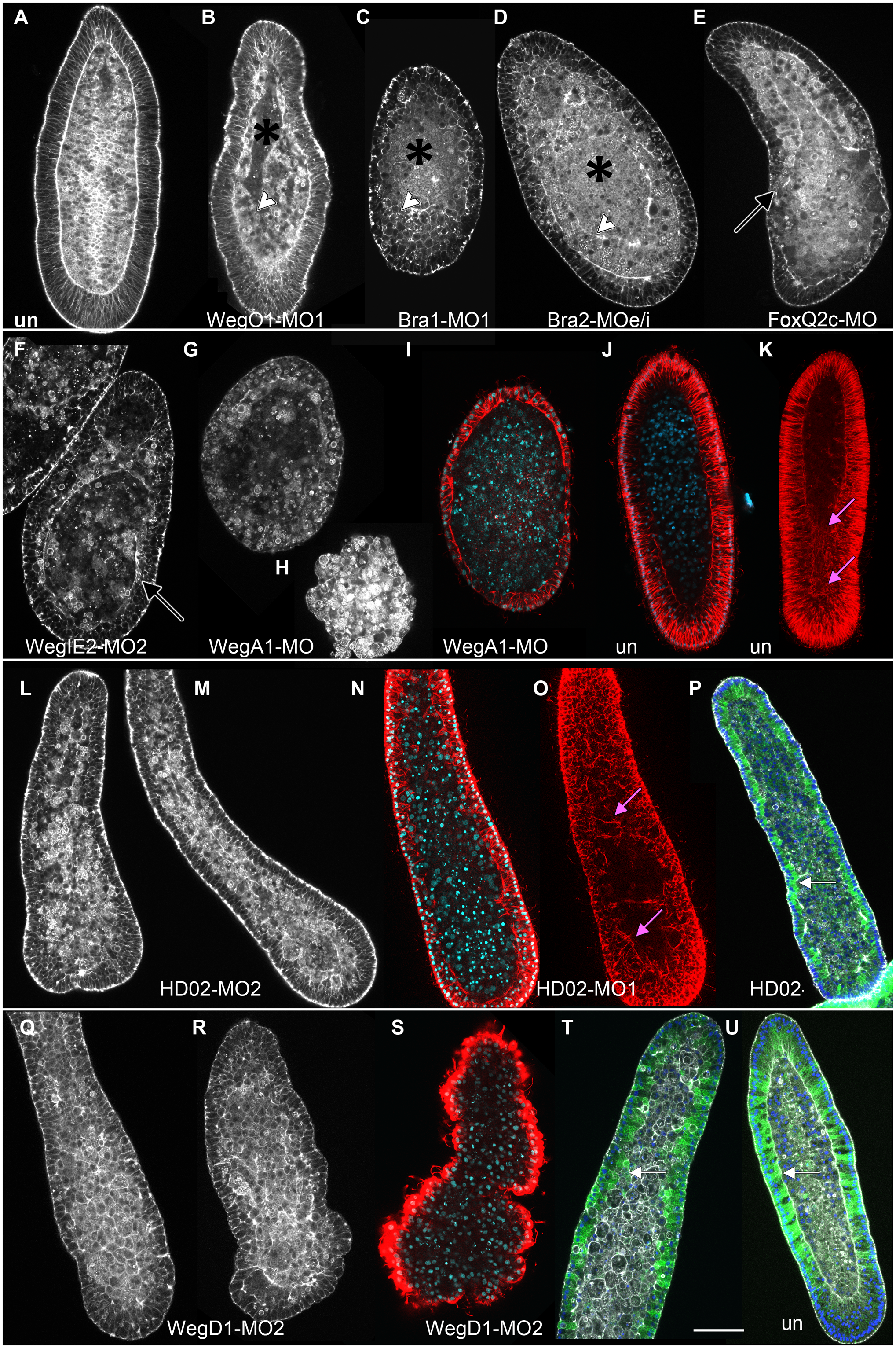

Supplement: File S8 — Confocal images of control and morpholino embryos fixed after about 48 hpf and phalloidin stained for the visualisation of cell contours (A-H, L, M, P-R) or anti-tubulin immunofluorescence (red) and nuclear staining with Hoechst or TOPRO-3 dyes (blue) (I-K, N, O, S). Endogenously expressed GFP-1 [92] provided a marker of the planula ectoderm (green in P, T, U). Oral poles are at the top in all images. Uninjected planula larvae (A) showed well-defined, epithelialized ectoderm and endodermal layers. WegO1 (B), Bra1 (C) and Bra2 (D) showed deficits of endoderm with regions of empty blastocoel still found (asterisks) as well as residual ectodermal cells accumulating towards the aboral pole (white arrowheads). This defect was much less severe in WegO1-MO embryos. FoxQ2c-MO (E) and WegIE2-MO (F) embryos showed severely disrupted endodermal layers (black arrows). WegA1-MO injection generated aggregates of endodermal-like cells of variable sizes covered in some (G, I) but not all (H) cases by a thin layer of ectodermal cells. HD02-MO (L-P) and WegD1-MO (Q-T) embryos had severely disrupted morphology characterized by a disruption of the basal lamina between the endoderm and GFP-expressing ectoderm layers (white arrows in P and T; compare with the uninjected planula in U). The WegD1-MO embryos were filled with disorganized cell sheets and lacked the central stripe of cell destruction characteristic of normal endodermal cavity formation. Anti-tubulin staining revealed disorganized bundles of neurite-like processes in the ectoderm-endoderm interface in HD02-MO embryos contrasting with the more orderly organization in uninjected embryos (pink arrows in confocal images in O and K respectively, acquired at this level) and the lack of this layer in WegD1-MO embryos (S). Scale bar 50 µm. (TIF) [file pgen.1004590.s008.tif]
